# Supplementary material for: Undifferentiated Sarcomas Develop through Distinct Evolutionary Pathways
Source: Cancer Cell. 2019 Mar 18;35(3):441–456.e8. doi: 10.1016/j.ccell.2019.02.002 (PMC6428691; doi:10.1016/j.ccell.2019.02.002)
Supplement: Doucment S1. Figures S1–S7 and Table S6 [file mmc1.pdf]

Cancer Cell, Volume *35*

Supplemental Information

Undifferentiated Sarcomas Develop through Distinct Evolutionary Pathways

Christopher D. Steele, Maxime Tarabichi, Dahmane Oukrif, Amy P. Webster, Hongtao Ye, Matthew Fittall, Patrick Lombard, Iñigo Martincorena, Patrick S. Tarpey, Grace Collord, Kerstin Haase, Sandra J. Strauss, Fitim Berisha, Heli Vaikkinen, Pawan Dhami, Marnix Jansen, Sam Behjati, M. Fernanda Amary, Roberto Tirabosco, Andrew Feber, Peter J. Campbell, Ludmil B. Alexandrov, Peter Van Loo, Adrienne M. Flanagan, and Nischalan Pillay

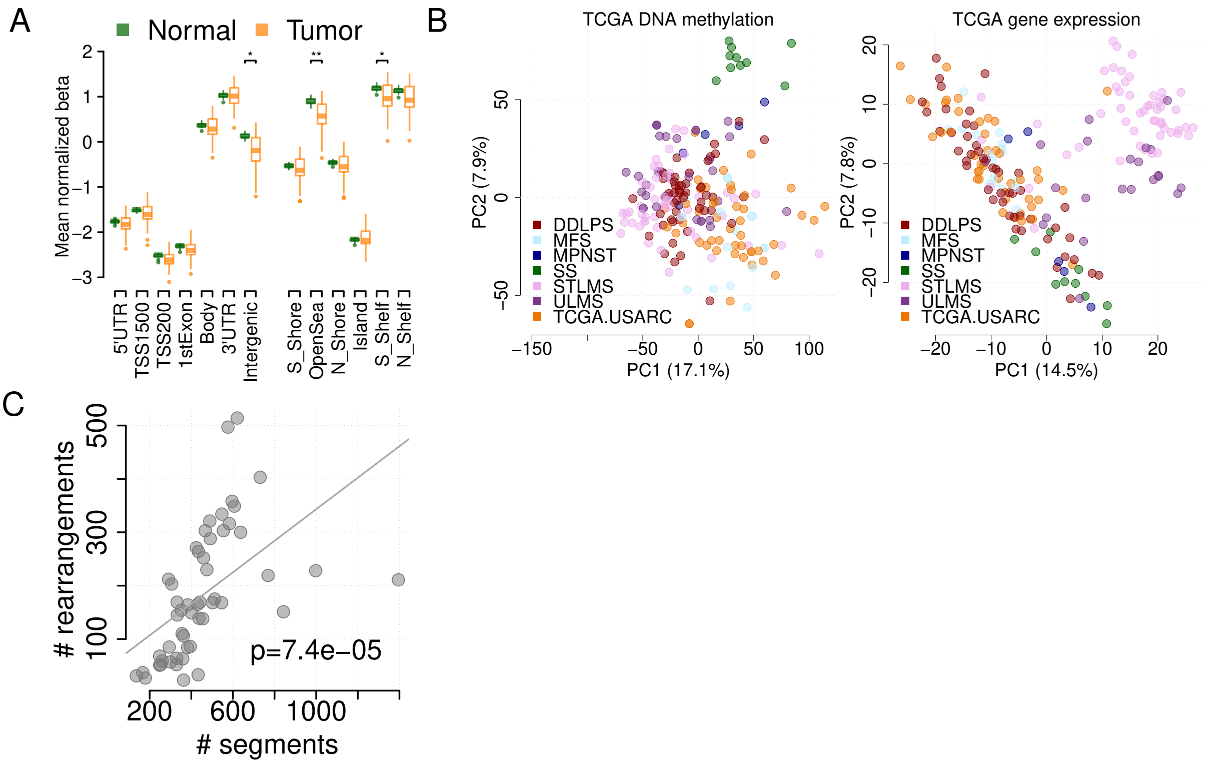

Figure S1. Methylation and RNA patterns in TCGA sarcoma cohort. Related to Figure 1.

(A) Methylation profiling demonstrated large differences between TCGA USARC (orange) and our cohort adjacent normal tissue (green). The pattern of predominant hypomethylation in intergenic and open sea regions observed in USARC was recapitulated here. Boxes show lower quartile, median and upper quartile; lines denote furthest point within 1.5x the interquartile range away from the box; points denote data further than 1.5x the interquartile range away from the box.

(B) Principal components analysis of DNA methylation data (left) or gene expression data ($\log_{10}$(FPKM+1), right) from all sarcoma subtypes in TCGA. Methylation PCs distinguish synovial sarcoma from all other subtypes, and fails to identify distinct USARC subgroups, while RNA PCs distinguish uterine and soft-tissue leiomyosarcoma from all other subtypes, and fail to identify distinct USARC subgroups.

(C) The number of copy number alterations (number of segments) and number of rearrangement breakpoints are strongly correlated (linear regression, β_1=0.3, $p<0.001$).

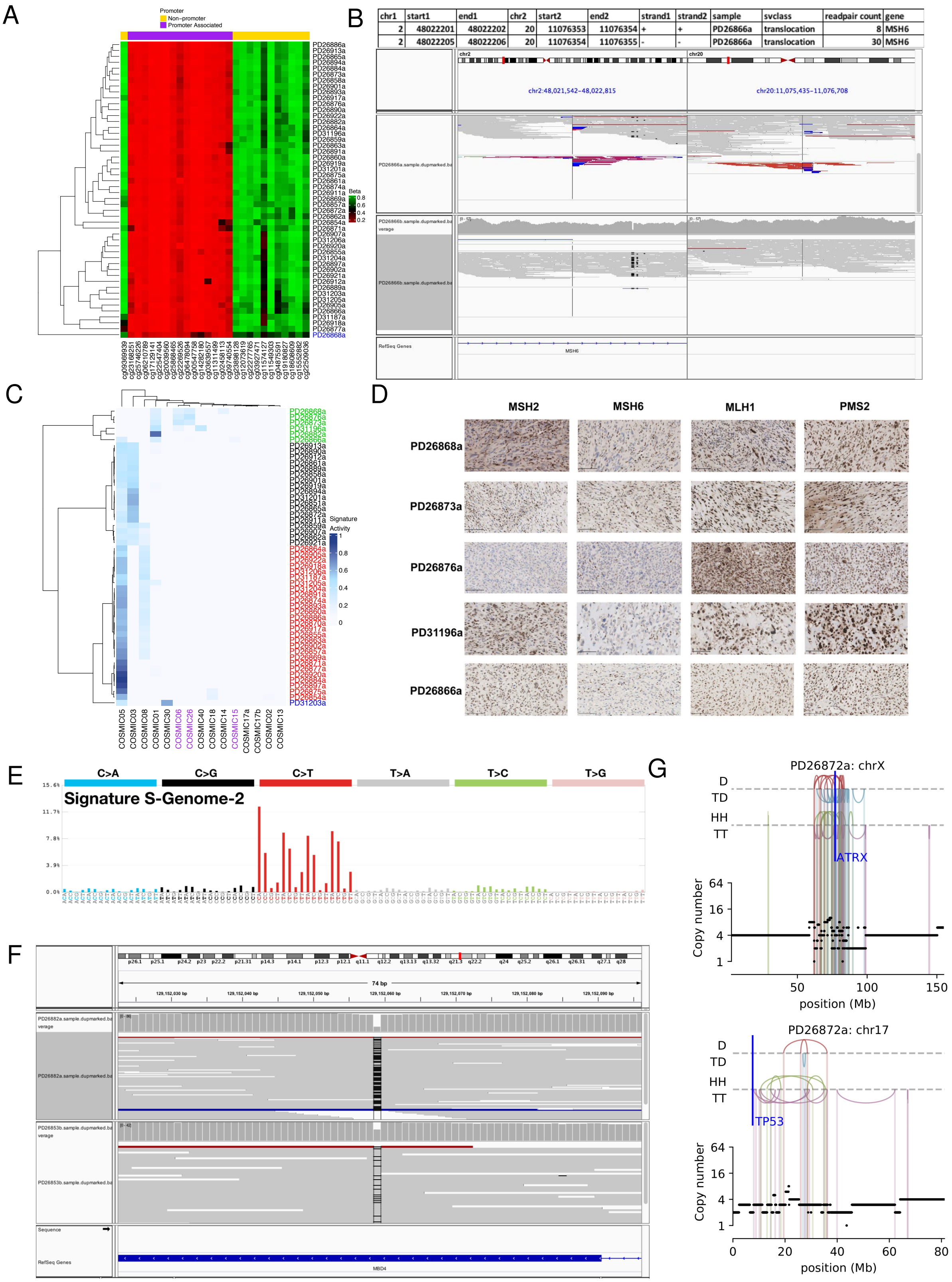

Figure S2. Mismatch repair deficiency and hypermutation in USARC. Related to Figure 1.

(A) Heatmap of beta values of EPIC methylation probes overlapping *MSH2*. PD26868a (blue text) shows relative promoter hypermethylation and gene body hypomethylation compared to all other samples, suggesting *MSH2* silencing through epigenetic regulation.

(B) IGV plot of *MSH6* translocation t(2;20) breakpoints that are predicted to be disruptive.
(C) Mutational signature heatmap. Cosmic mismatch repair deficiency signatures are shown in purple text. Samples are clustered into four groups; hypermutators (green text), homologous recombination deficient (black text), unknown aetiology (red text) and *NTHL1* deficient (blue text).
(D) Mismatch repair protein immunohistochemistry. *MSH2*, *MSH6*, *MLH1* and *PMS2* immunohistochemistry corroborate gene sequencing, mutational signature and methylation findings. Concordant loss of protein expression of *MSH2* and *MSH6* in tumor cells is seen in PD26868a, PD26873a, PD26876a and PD31196a. The *NTHL1* deficient tumor (PD31203a) shows retention of all four mismatch repair proteins. Normal inflammatory cells serve as internal control. Scalebar:100μm.
(E) Sequence context of spectra of mutations (predominantly C>T transitions) indicative of COSMIC signature 30 in sample PD31203a.
(F) PD26882a: IGV browser plot of *MBD4* showing a heterozygous DNA glycosylase domain frameshift mutation (p.L482Wfs*9) in the germline (lower) with loss of heterozygosity in the tumor sample (upper).
(G) Chromothriptic events are observed in regions with key driver genes. Individual rearrangements are shown in top panel (D=deletion, TD=tandem duplication, HH and TT=head-head and tail-tail inversion respectively), total copy number is shown in bottom panel. Genes of interest are highlighted in blue. Translocations are not displayed.

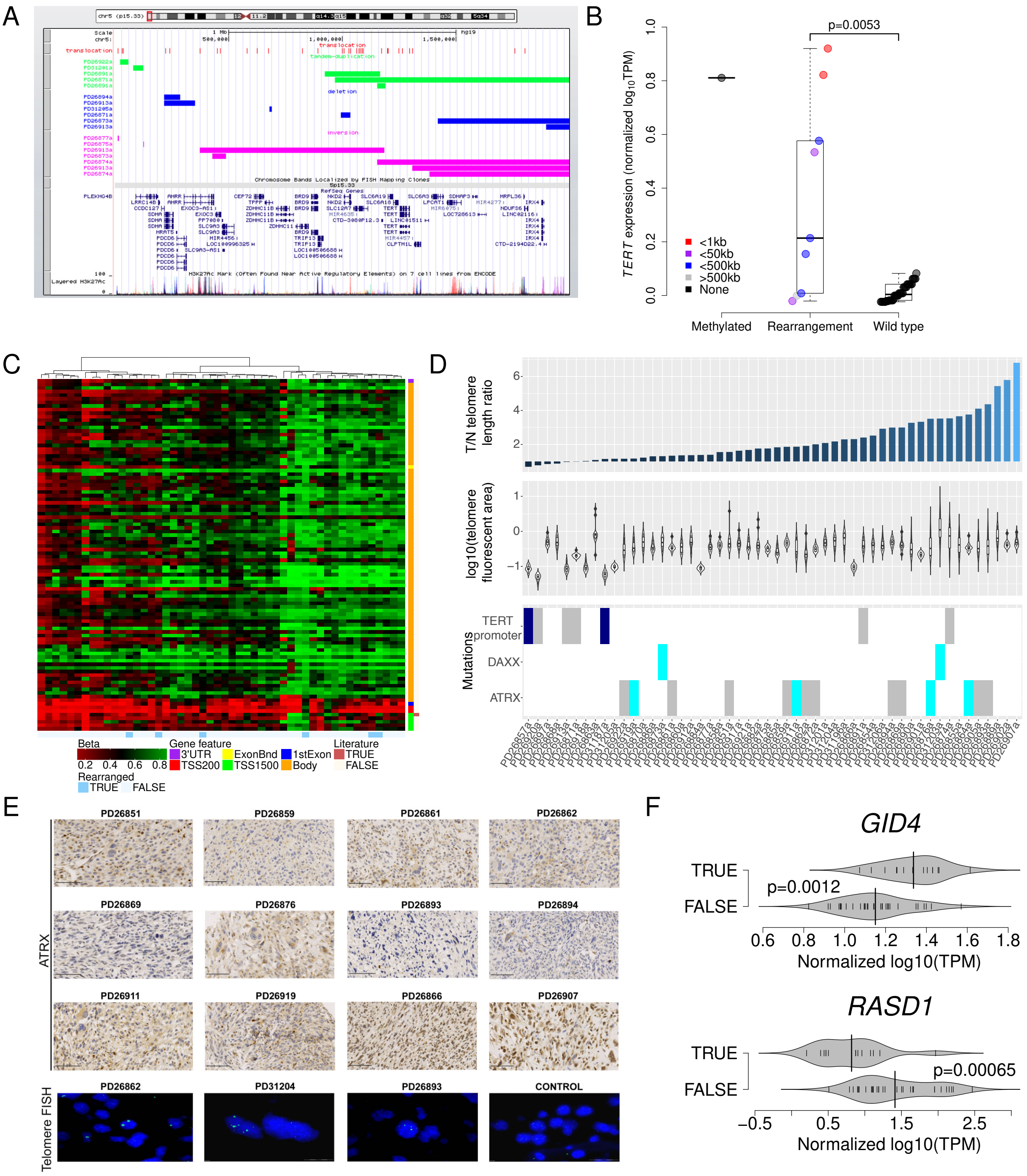

Figure S3. Telomere maintenance pathway. Related to Figure 1.

(A) Browser plot demonstrating the number and spectrum of rearrangements in proximity to *TERT*.

(B) Boxplot of *TERT* expression in samples that have promoter hypermethylation of cg11625005 (Methylated), a rearrangement within 100kb of *TERT* (Rearrangement) or no alterations in *TERT* (Wild Type). Color indicates the distance between the non-TERT rearrangement partner and its closest muscle-specific enhancer. Samples with a rearrangement within 100kb of *TERT* have increased expression of *TERT*, with those that colocalise *TERT* closest to a muscle-specific enhancer (red) having the strongest expression of *TERT*, suggesting activation of *TERT* through enhancer

hijacking. Boxes show lower quartile, median and upper quartile; lines denote furthest point within 1.5x the interquartile range away from the box.

(C) Methylation of probes surrounding *TERT*. X-axis=samples, y-axis=probes. Probe cg11625005 (brown annotation) in PD26857a and PD31187a show relative hypermethylation of the commonly epi-mutated probe cg11625005 in the *TERT* promoter region. Samples with a blue annotation harbor a rearrangement within 100kb of *TERT*.

(D) Tumor:normal telomere ratio estimated from whole genome sequencing. 88.4% of tumor samples show relative telomere lengthening compared to matched normal tissue (upper panel). Violin plots with integrated boxplots indicate telomere FISH analysis data where fluorescent signal area was measured in a minimum of 10 cells (middle panel). *ATRX* and *DAXX* mutations are associated with telomere lengthening (bottom panel). Blue box: promoter epimutation. Grey box: rearrangement. Aqua box: single nucleotide variant or indel.

(E) *ATRX* immunohistochemistry results confirm that *ATRX* mutant samples show loss of protein expression in tumor cells. Representative *ATRX* wildtype samples demonstrating retained nuclear *ATRX* expression (PD26866a, PD26907a). Scalebar 100μm. Representative FISH images of telomere fluorescence demonstrating large, bright signals in tumor cells. Testicular tissue used as a control demonstrates the difference in telomere signal size and intensity.

(F) Genes in the 17p11.2 region that have significantly correlated gene expression and rearrangement status.

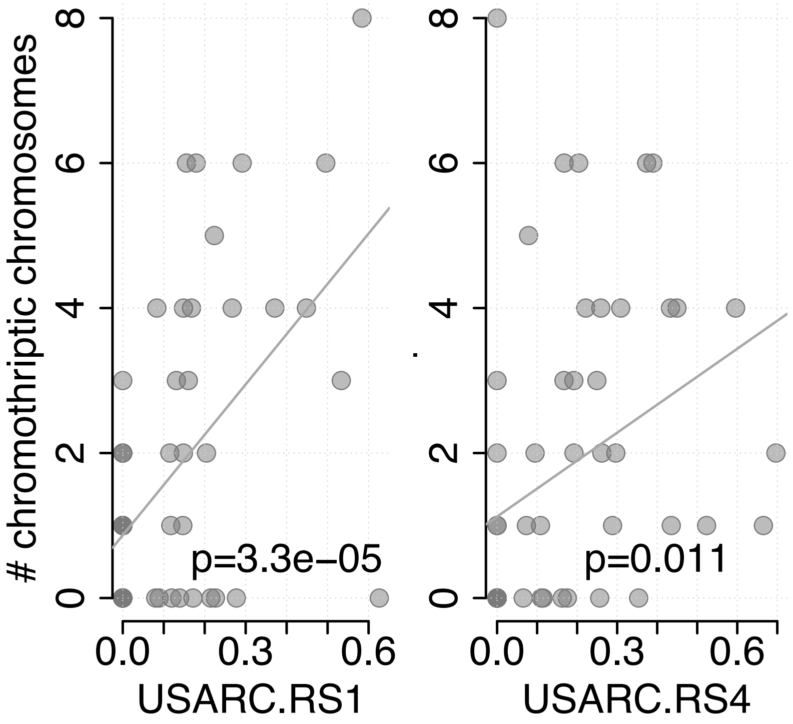

Figure S4. USARC genomic instability correlates. Related to Figure 4.
The number of chromothriptic chromosomes is positively correlated with both the exposure to USARC.RS1, and CNS5.

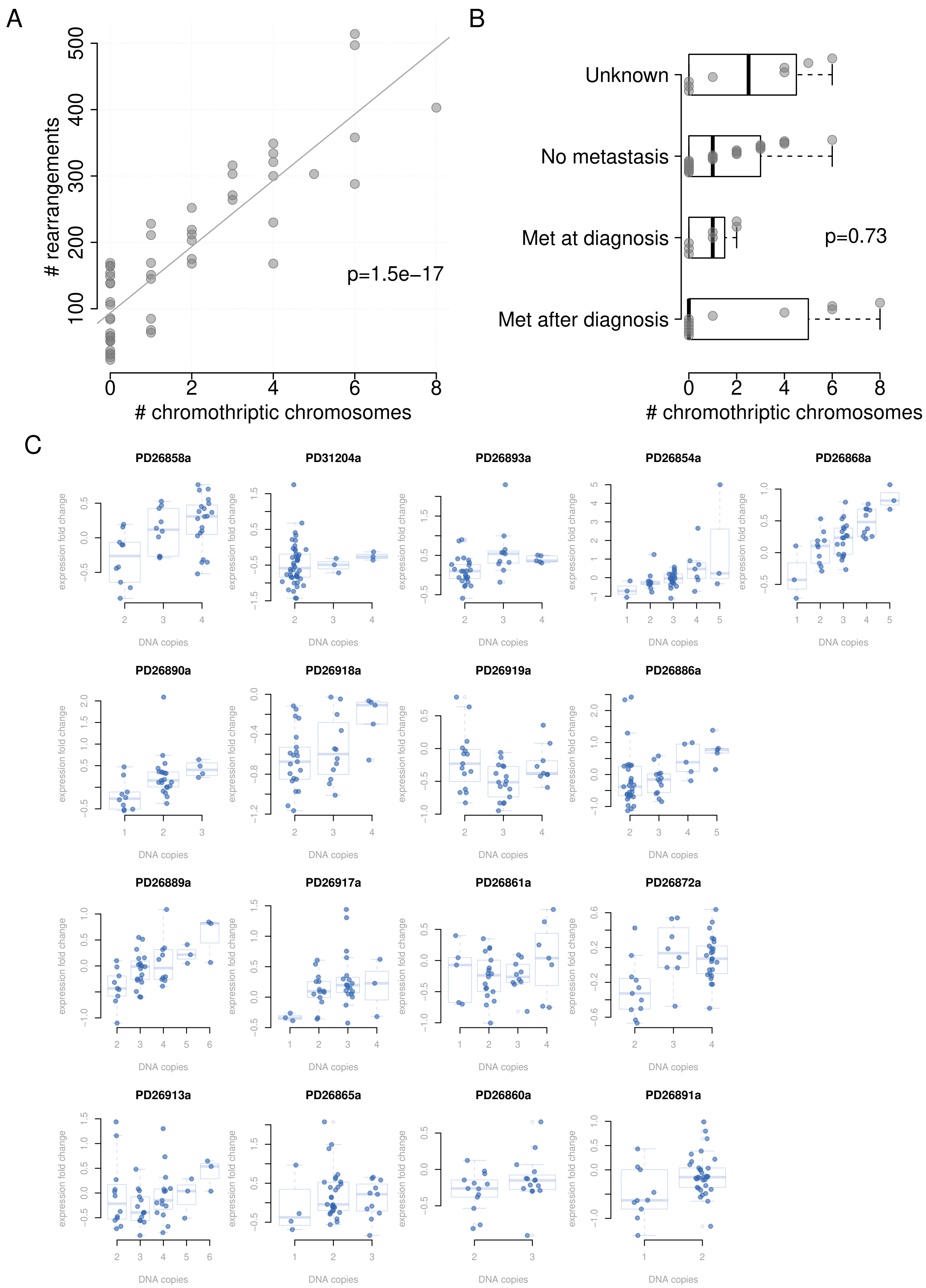

Figure S5. Copy number has a direct effect on gene expression. Related to Figure 5.

(A) The number of chromothriptic chromosomes is correlated with the number of rearrangements and is enriched in the rearrHi group (samples with > 100 rearrangements).

(B) There is no effect of chromothriptic chromosomes on metastasis status (ANOVA, p=0.4). Boxes show lower quartile, median and upper quartile; lines denote furthest point within 1.5x the interquartile range away from the box.

(C) Gene expression fold change compared to all other samples (y-axis) dependent on copy number (x-axis) for multiple samples (panels) displays a trend of increasing gene expression with increasing copy number.

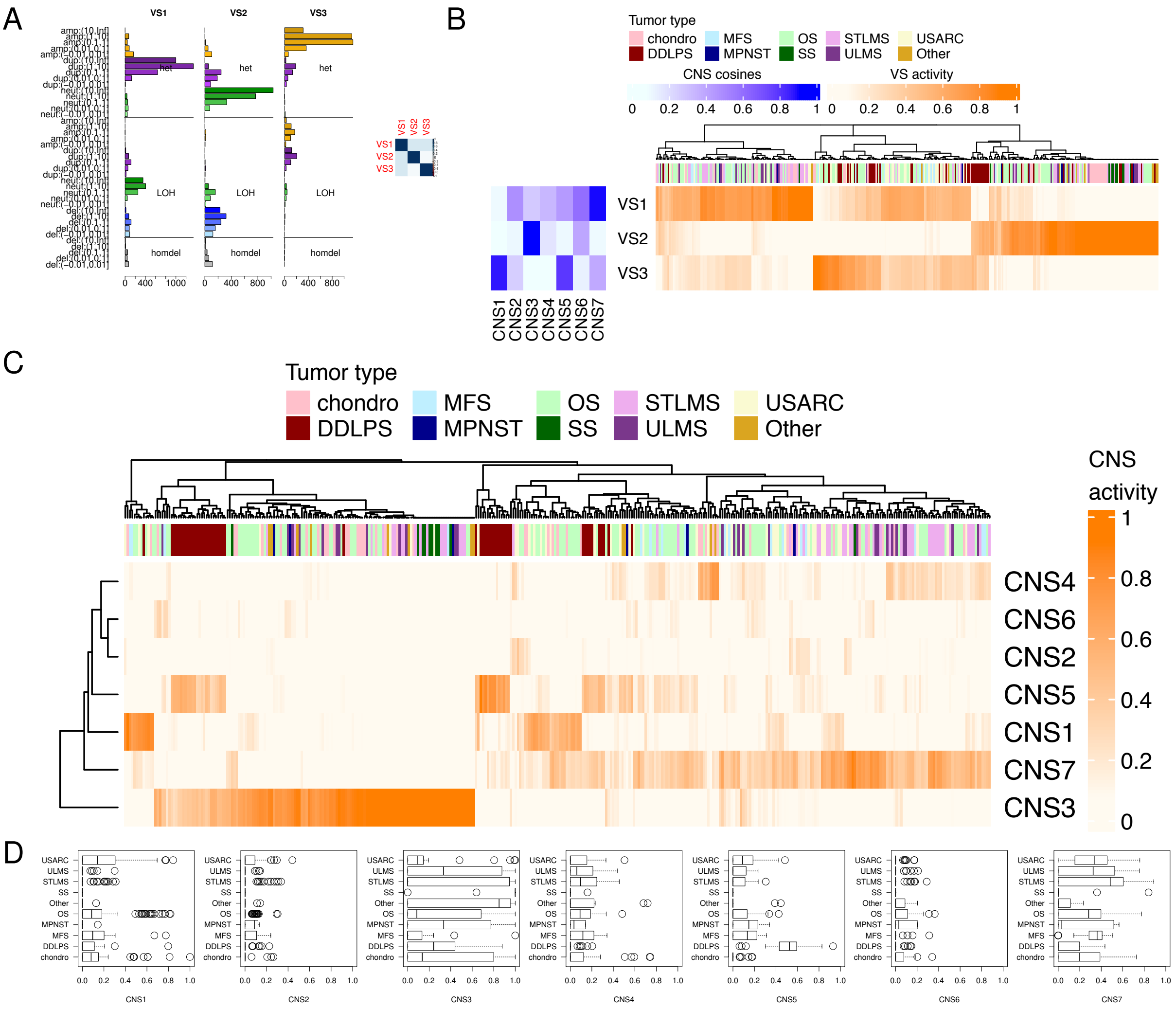

Figure S6. Copy number signature validation. Related to Figure 5.

(A) Three copy number signatures identified in 320 sarcomas of multiple subtypes; 43 chondrosarcoma (chondro), 51 dedifferentiated liposarcoma (DDPLS), 17 myxofibrosarcomas (MFS), 6 malignant peripheral nerve sheath tumor (MPNST), 112 osteosarcoma (OS), 10 synovial sarcoma (SS), 52 soft tissue leiomyosarcoma (SFLMS), 27 uterine leiomyosarcoma (ULMS), 43 undifferentiated pleomorphic sarcoma (UPS), and 12 assorted sarcomas (Other).

(B) Activities of each validation signature in the validation cohort per sample. Cosine similarities between USARC signatures (CNS1-7) and validation signatures (blue heatmap). VS1-3 have all been identified in the USARC cohort.

(C) Predicted activities of CNS1-7 in the validation cohort (TCGA samples and other non-USARC sarcomas) using deconstructSigs.

(D) Boxplot of predicted activities of CNS1-7 in each tumor type. UPS – undifferentiated pleomorphic sarcoma, ULMS – uterine leiomyosarcoma, STLMS – soft tissue leiomyosarcoma, SS – synovial sarcoma, OS – osteosarcoma, MPNST – malignant peripheral nerve sheath tumor, MFS – myxofibrosarcoma, DDLPS – dedifferentiated liposarcoma, chondro – chondrosarcoma, Other – variety of low grade spindle cell sarcomas. Boxes show lower quartile, median and upper quartile; lines denote furthest point within 1.5x the interquartile range away from the box; points denote data further than 1.5x the interquartile range away from the box.

Table S6 – Samples amenable to mutational and genome-duplication timing in the USARC and TCGA datasets. Related to Figure 7.

	USARC	TCGA
Timed	47 (10 CNsig1; 1 CNsig2; 0 CNsig3; 7 CNsig4; 7 CNsig5; 4 CNsig6; 18 CNsig7)	84 (6 CNsig1; 1 CNsig2; 1 CNsig3; 5 CNsig4; 19 CNsig5; 52 CNsig7)
Not timeable	6 (6 CNsig 3)	8 (8 CNsig 5)
Ploidy disagreement	-	47
Signatures not available	-	10

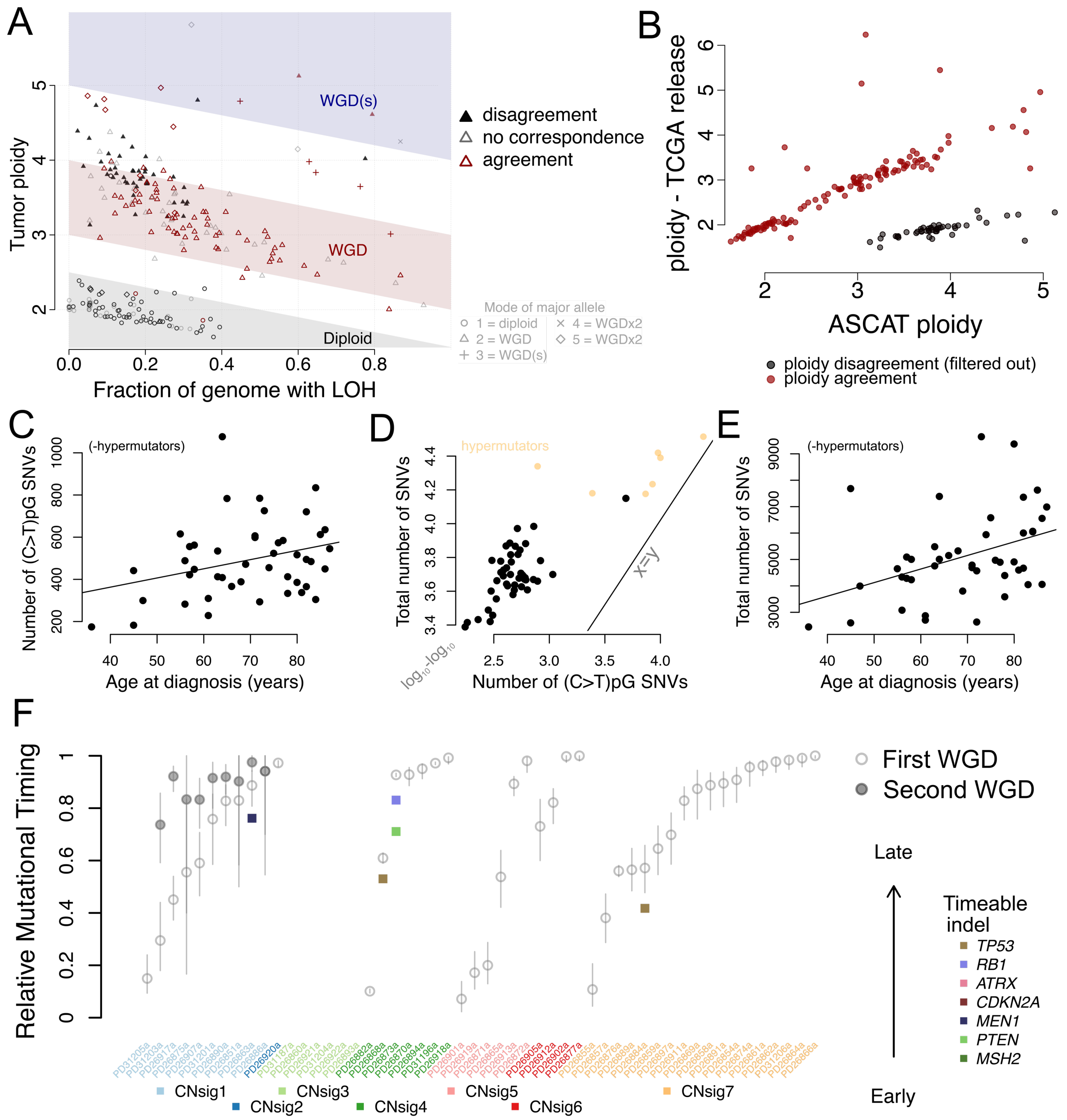

Figure S7. Timing of mutations and genome doubling events. Related to Figure 7.

(A) Ploidy/LOH space for TCGA cohort. Points are colored by whether the ASCAT estimate of the ploidy agrees with that from ABSOLUTE published by TCGA, whereas the shape of the data points relates to their ploidy and represents the mode of the major allele.

(B) TCGA ABSOLUTE ploidy against ASCAT ploidy. Only those samples in agreement (red) were taken further for timing analysis.

(C) Linear relationship between number of (C>T)pG SNVs and age at diagnosis.

(D) Relationship between total number of SNVs and number of (C>T)pG SNVs in samples. Equality line is shown. The gradient of a linear fit is similar to the equality gradient, indicating that timing will be similar using either total SNVs or only (C>T)pG SNVs.

(E) Linear relationship between total number of SNVs and age at diagnosis.

(F) Timing of genome doubling events and driver mutations in the USARC cohort using only (C>T)pG SNVs. Confidence intervals are larger than when using all SNVs (see Figure 5), but the broad picture remains similar. MRCA – most common recent ancestor. Vertical bars: 95% confidence intervals.
