# Supplementary material for: Undifferentiated Sarcomas Develop through Distinct Evolutionary Pathways
Source: Cancer Cell. 2019 Mar 18;35(3):441–456.e8. doi: 10.1016/j.ccell.2019.02.002 (PMC6428691; doi:10.1016/j.ccell.2019.02.002)
Supplement: Document S2. Article plus Supplemental Information [file mmc7.pdf]

Cancer Cell

Article

Undifferentiated Sarcomas Develop through Distinct Evolutionary Pathways

Graphical Abstract

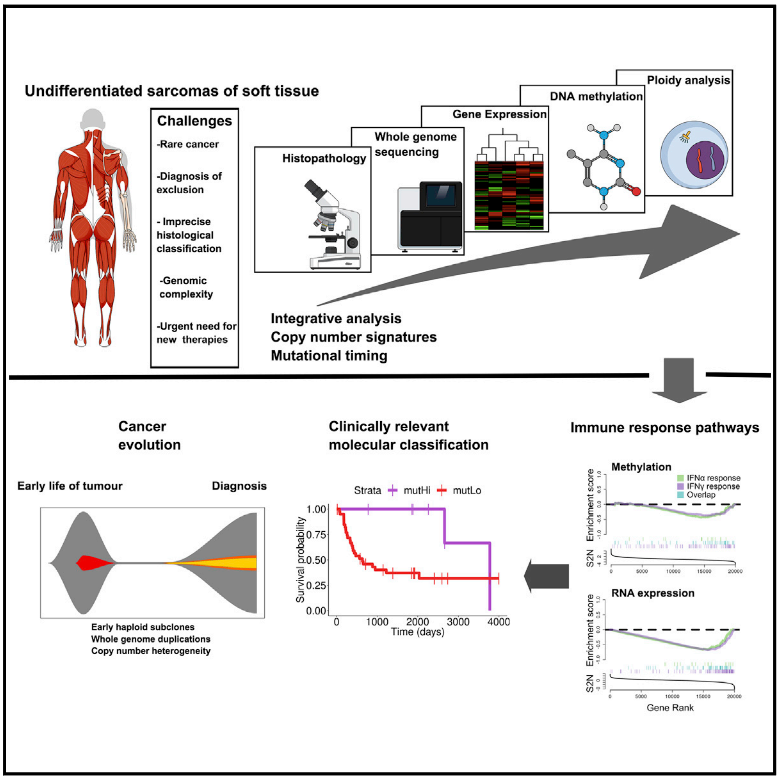

Highlights

- Undifferentiated sarcomas contain biologically relevant molecular subgroups
- Identification of mismatch repair deficiency open up alternate avenues for therapy
- Pseudohaploidization is a recurrent event in undifferentiated sarcomas
- Copy-number signatures are useful for inferring states of sarcoma evolution

Authors

Christopher D. Steele, Maxime Tarabichi, Dahmane Oukrif, ..., Peter Van Loo, Adrienne M. Flanagan, Nischalan Pillay

Correspondence

n.pillay@ucl.ac.uk

In Brief

Steele et al. determine the molecular landscape of undifferentiated sarcomas. They identify tumors with high mutation burdens, which are enriched for activation of immune pathways and have good prognoses, and deduce four tumorigenic routes, all of which begin with driver mutations before whole genome duplication.

Steele et al., 2019, Cancer Cell *35*, 441–456
March 18, 2019 © 2019 The Author(s). Published by Elsevier Inc.
https://doi.org/10.1016/j.ccell.2019.02.002

Undifferentiated Sarcomas Develop through Distinct Evolutionary Pathways

Christopher D. Steele,[1] Maxime Tarabichi,[2] Dahmane Oukrif,[1] Amy P. Webster,[3] Hongtao Ye,[4] Matthew Fittall,[2] Patrick Lombard,[1] Iñigo Martincorena,[5] Patrick S. Tarpey,[5] Grace Collord,[5] Kerstin Haase,[2] Sandra J. Strauss,[1,6] Fitim Berisha,[4] Heli Vaikkinen,[7,8] Pawan Dhami,[7] Marnix Jansen,[1,9] Sam Behjati,[5,10] M. Fernanda Amary,[4] Roberto Tirabosco,[4] Andrew Feber,[11] Peter J. Campbell,[5,12] Ludmil B. Alexandrov,[13] Peter Van Loo,[2,14] Adrienne M. Flanagan,[1,4] and Nischalan Pillay[1,4,15,*]

[1]Research Department of Pathology, Cancer Institute, University College London, London WC1E 6BT, UK
[2]Cancer Genomics Laboratory, The Francis Crick Institute, London NW1 1BF, UK
[3]Department of Cancer Biology, UCL Cancer Institute, University College London, London, UK
[4]Department of Cellular and Molecular Pathology, Royal National Orthopaedic Hospital NHS Trust, Stanmore, Middlesex HA7 4LP, UK
[5]Cancer Genome Project, Wellcome Trust Sanger Institute, Wellcome Trust Genome Campus, Hinxton, Cambridgeshire CB10 1SA, UK
[6]Department of Oncology, University College London Hospital NHS Foundation Trust, London, NW1 2PG, UK
[7]Genomics and Genome Engineering Core Facility, CRUK-UCL Centre, Cancer Institute, University College London, London WC1E 6BT, UK
[8]Research Department of Oncology, Cancer Institute, University College London, London WC1E 6BT, UK
[9]Department of Cellular Pathology, University College London Hospital NHS Foundation Trust, London NW1 2BU, UK
[10]Department of Paediatrics, University of Cambridge, Cambridge CB2 0QQ, UK
[11]Department of Targeted Intervention, Division of Surgery and Interventional Science, University College London, London WC1E 6BT, UK
[12]Department of Haematology, University of Cambridge, Hills Road, Cambridge CB2 2XY, UK
[13]Department of Cellular and Molecular Medicine, University of California, San Diego 92093, USA
[14]Department of Human Genetics, University of Leuven, 3000 Leuven, Belgium
[15]Lead Contact
*Correspondence: n.pillay@ucl.ac.uk
https://doi.org/10.1016/j.ccell.2019.02.002

SUMMARY

Undifferentiated sarcomas (USARCs) of adults are diverse, rare, and aggressive soft tissue cancers. Recent sequencing efforts have confirmed that USARCs exhibit one of the highest burdens of structural aberrations across human cancer. Here, we sought to unravel the molecular basis of the structural complexity in USARCs by integrating DNA sequencing, ploidy analysis, gene expression, and methylation profiling. We identified whole genome duplication as a prevalent and pernicious force in USARC tumorigenesis. Using mathematical deconvolution strategies to unravel the complex copy-number profiles and mutational timing models we infer distinct evolutionary pathways of these rare cancers. In addition, 15% of tumors exhibited raised mutational burdens that correlated with gene expression signatures of immune infiltration, and good prognosis.

INTRODUCTION

Undifferentiated sarcomas (USARCs) of adults are soft tissue tumors that are among the most karyotypically complex of all cancers (TCGA, 2017). These tumors were previously known as malignant fibrous histiocytomas but historically have had multiple designations based on advances in diagnostic criteria (Fletcher, 2014). They are diagnosed by exclusion of other sarcoma entities and likely represent a final common morphological endpoint of a variety of sarcomas and, possibly, other tumors (Fletcher et al., 2001). Prognosis for these patients is poor, with a median survival for those with advanced, metastatic disease of approximately 12 months (Savina et al., 2017). The benefit of systemic therapy, particularly in the adjuvant setting is controversial (Linch et al., 2014). A lack of objective diagnostic criteria has led to a dearth of studies interrogating genomic complexity

Significance

USARC is not a specific tumor entity but rather a "wastepaper basket" grouping of sarcomas that cannot be classified. There are limited therapeutic options for patients and the biology underlying USARC tumorigenesis remains poorly understood. We show that a genomic classification for USARC is clinically and biologically relevant. Deconvolution of the complex copy number and rearrangement landscapes highlight USARC as an exemplar model to study chromothripsis, early haploidy, and WGD events in cancer. We also show that these tumorigenic pathways are active to different degrees in other sarcoma subtypes from The Cancer Genome Atlas, shedding light on pan-sarcoma mechanisms of tumorigenesis.

This is an open access article under the CC BY license (http://creativecommons.org/licenses/by/4.0/).

in these tumors at base pair resolution. The ability to probe this complexity is important because genomic instability is a key catalyst in cancer evolution, fuels tumor heterogeneity, and is relevant therapeutically (Burrell et al., 2013). The karyotypic complexity inherent in USARCs also suggests that interrogating and distilling structural aberrations in these tumors may yield large returns in our understanding of the disease.

Recent work from The Cancer Gene Atlas (TCGA) has characterized the cancer driver gene landscape of a number of soft tissue sarcoma types, including 44 undifferentiated pleomorphic sarcomas (henceforth TCGA.USARC) using a multi-omic approach (TCGA, 2017). Here, we sought to extend that work through whole genome sequencing (WGS), resulting in insights into USARC biology and evolution and potential avenues for treatment.

RESULTS

A collection of 76 tumor samples diagnosed using standard of care were selected for investigation, based on availability of adequate nucleic acid, and were required to be radio- and chemotherapy naive in order to ensure high-quality, tumor-rich specimens and to avoid confounding by prior treatment. Eight cases were reclassified as other sarcoma entities through pathological review or genomic characterization and thus excluded (STAR Methods and Figure 1A). Tumors were classified into morphological variants (pleomorphic, spindle, epithelioid, or mixed) according to the most recent guidelines (WHO, 2013) for further analysis (Figure 1B).

USARCs Are Characterized by Relative Global Hypomethylation Compared with Normal Muscle

Biologically relevant subgroups of mesenchymal tumors have recently been identified through DNA methylation profiling (Rohrich et al., 2016). These molecular classification schemes hold great promise for sarcoma clinical diagnostics and add value to the traditional histological classification for prognostication. To determine if molecularly defined subgroups could be identified in USARCs, we performed genome-wide methylation profiling (EPIC array) and gene expression analysis (RNA sequencing, Table S1) for all tumor samples for which sufficient high-quality nucleic acid was available. Compared with adjacent normal tissues (skeletal muscle) USARC methylomes showed relative hypomethylation on a genome-wide scale with the majority of the signal confined to intergenic regions of the genome and in "open sea" regions rather than in promoter regions or CpG islands (Figure 1C), and this result was reproduced in the TCGA.USARC dataset (Figure S1A).

Principal-component analysis of both the methylation and gene expression data revealed strong separation between tumor and normal samples; however, it failed to delineate any clear subgroups within USARCs (Figure 1D), and, similarly TCGA.USARC are heterogeneous and do not represent a distinct sarcoma subtype at the DNA methylation level (Figure S1B). Using unsupervised hierarchical clustering we also found that there was poor concordance between the methylation and the gene expression sample clusters at a global level (Figure 1D), reflecting the finding that global changes in methylation were more prominent in non-genic regions. In addition, there were no associations with histological subtype.

Both Mutational and Rearrangement Burden Are Characteristics of Genomic Complexity in USARCs

We then analyzed 52 of the USARC samples using WGS, which were sequenced to an average depth of 70× along with DNA from blood from the same patients sequenced to 30× depth (Table S1). Somatic variant calling was performed using a validated suite of software and bespoke post-processing filters. Per sample, the median number of single nucleotide variants (SNVs) was 4,741 (range: 2,164–32,108) (Table S1), of indels was 449 (range: 180–20,073) (Table S1), and of rearrangements was 166 (range: 23–514) (Table S1). A further extension cohort of 16 samples of USARCs (Table S1) were sequenced to a mean target depth of 403× on a cancer gene exome panel covering 3 Mb of the genome, including intronic coverage of *TP53*, *RB1*, *ATRX*, and *CDKN2A*.

USARCs present a relatively low median SNV/indel mutational burden and a high median number of rearrangements and resulting copy-number alterations (Figure S1C). However, a subgroup presented with high SNV/indel and low rearrangement burdens. We thus classified USARCs into three molecular subgroups: mutation high—rearrangement low (mutHi-rearrLo); mutation low—-rearrangement high (mutLo-rearrHi); and mutation low—rearrangement low (mutLo-rearrLo; Figure 1E, Table S1). Of the 45 tumors demonstrating 15,000 or fewer SNV/indel mutations across the genome, 33 patients harbored 100 or more rearrangements per tumor (mutLo-rearrHi group). In contrast, 7 tumors demonstrated a hypermutator phenotype with a minimum of 15,000 SNV/indel mutations per tumor (>5 mutations per Mb; median 28,370), all of which had a relatively low rearrangement burden (median 68) (mutHi-rearrLo group). The third molecular subgroup consisted of 12 tumors with modest rearrangement and mutational burdens in comparison with the others (mutLo-rearrLo). Extension samples were classified as mutation high (tMutHi) or mutation low (tMutLo).

Mismatch Repair Deficiency in the Muthi-rearrLo USARC Subgroup

The finding that ~13% (n = 7) of USARC samples had an elevated mutational burden prompted further investigation. We found somatic driver SNVs within *MSH2* in two cases (PD26873a and PD26876a), both with somatic copy-number loss of the wild-type allele (Table S1). However, we also observed aberrations in *MSH2*, including promoter methylation (PD26868a; Figure S2A) and a predicted disruptive translocation on the forward strand in intron two associated with loss of heterozygosity (LOH) (PD26866a Figure S2B). A fifth patient (PD31196a) was found to have a pathogenic germline mutation in *MSH6* (p.V878A) with somatic loss of the wild-type allele in the tumor. All five of these tumors exhibited mutational signatures of mismatch repair (MMR) deficiency (signatures 6, 15, 26, and 40, Figure S2C) and protein loss of one or both of MSH2 and MSH6 (Figure S2D).

Sample PD31203a showed a mutational signature (signature 30) that strongly matched the base excision repair *NTHL1* deficiency pattern (Figure S2E), previously only seen in a breast cancer (Nik-Zainal et al., 2016) and an osteosarcoma (Behjati et al., 2017). A rare pathogenic germline heterozygous nonsense mutation of *NTHL1* (pQ90*) with somatic loss of the wild-type allele was confirmed in this patient. Finally, PD26882a showed more than 28,000 mutations with strong activity of signature 1

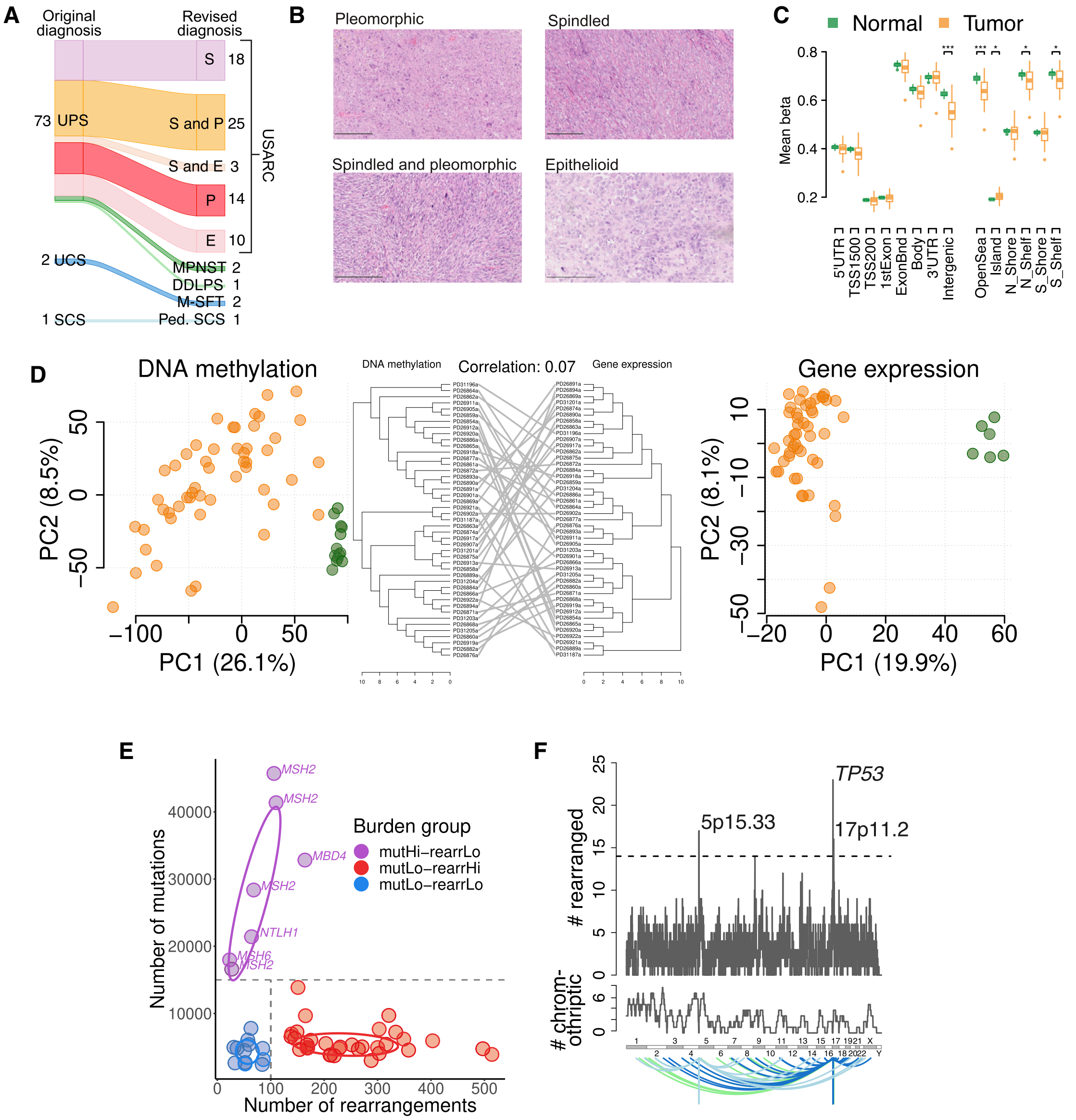

Figure 1. Molecular Classification of USARCs

(A) Alluvial diagram showing tumor diagnosis reclassification following expert pathological review. UPS, undifferentiated pleomorphic sarcoma; USARC, undifferentiated sarcoma; UCS, unclassified sarcoma; SCS, spindle cell sarcoma; S, spindle; P, pleomorphic; E, epithelioid; MPNST, malignant peripheral nerve sheath tumor; DDLPS, dedifferentiated liposarcoma; M-SFT, malignant solitary fibrous tumor; Ped. SCS, pediatric spindle cell sarcoma. Numbers indicate the number of samples for each subtype.

(B) H&E staining of four representative USARC subtypes. Scale bars, 250 μm.

(C) Mean methylation of probes categorized by genomic position (left) or position relative to CpG islands (right), in USARC samples (orange) and normal adjacent tissue (green); *$q < 0.05$, **$q < 0.01$, ***$q < 0.001$. Boxes show lower quartile, median and upper quartile; lines denote furthest point within 1.5× the interquartile range away from the box; points denote data further than 1.5× the interquartile range away from the box.

(D) Principal-component analysis of tumor (orange) and normal (green) samples for both methylation array data (left) and RNA sequencing data (right) as well as shared hierarchical clustering of RNA and methylation data (center).

(E) Scatterplot of rearrangement burden (x axis) against SNV/indel burden (y axis) of USARC samples from WGS. Samples were categorized into three groups: mutation high, rearrangement low (mutHi-rearrLo, purple), mutation low, rearrangement high (mutLo-rearrHi, red), and mutation low, rearrangement low (mutLo-rearrLo, blue). Decision boundary is shown as a dashed line. Filled circles are individual data points, ovals 50% probability intervals.

(F) Number of samples that have ≥1 rearrangement in genomic windows of 1 Mb (top), number of samples that have chromothriptic regions overlapping genomic windows of 1 Mb (middle), and rearrangement partners of rearrangements within regions that are significantly enriched (bottom). Regions with significant enrichment ($q < 0.2$) are labeled.

See also Figures S1–S3 and Table S1.

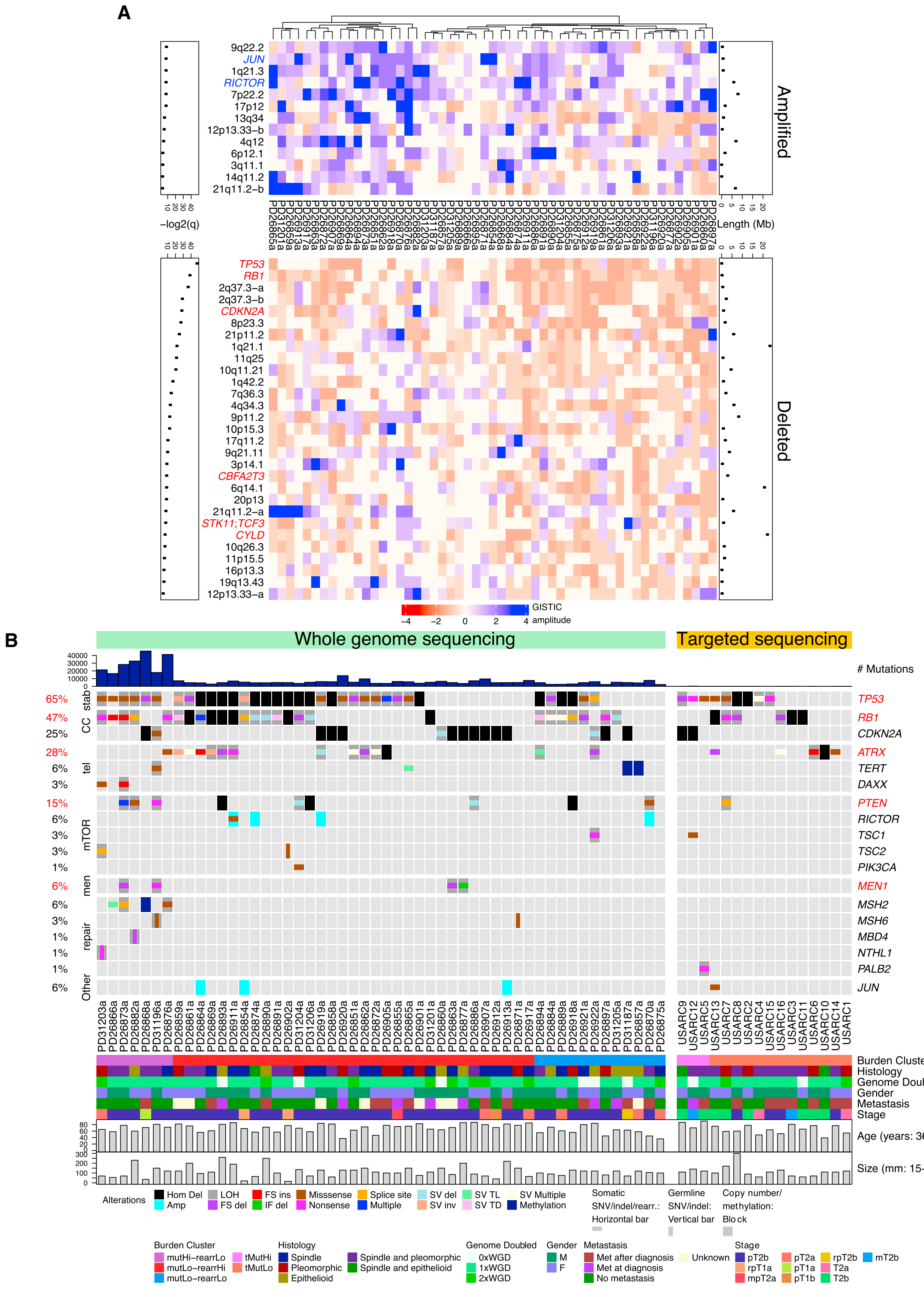

Figure 2. Integration of Driver Events in USARCs

(A) Heatmap showing significant recurrently amplified or deleted regions (GISTIC q < 0.1). Known cancer driver genes in amplified regions are labeled in blue and those in deleted regions are labeled in red. Lengths of significant regions (Mb) are indicated above the heatmap, with –log2(q values) below.

(legend continued on next page)

with almost pure C > T transitions in a CpG context, likely caused by spontaneous deamination of methylated cytosines (Alexandrov et al., 2018). Such a strong activity of signature 1 and without evidence of MMR deficiency raised the possibility of failure of repair of the deaminated cytosines. This was confirmed by the discovery of biallelic inactivation of the DNA glycosylase gene *MBD4* (Figure S2F). *MBD4* prevents mutability at CpG sites and is a binding partner of the MMR protein MLH1 (Bellacosa et al., 1999). To the best of our knowledge, this is the first description of defective MBD4-associated DNA repair in sarcomas.

TP53, *RB1*, *CDKN2A*, *ATRX*, and 5p15.33 (*TERT*) Rearrangements Are Recurrent

Leveraging the higher resolution of WGS, we called putative structural variants and scrutinized these in order to identify potential cancer driver gene events.

As fusion genes are characteristic of many sarcoma subtypes, we first looked for structural variants causing gene fusions. Results of these analyses suggest that oncogenic chimeric fusions are rare events in the pathogenesis of USARCs (Table S1). Conversely, for identification of recessive mechanisms, we sought truncating rearrangements and overlapped these regions with known recessive cancer genes (Forbes et al., 2017). We identified 51 recurrently rearranged genes, 9 of which are known tumor suppressor genes (Table S1). In particular, recurrent disruptive rearrangements were identified in *TP53*, *RB1*, and *ATRX*. Using a bespoke tool for chromothripsis identification we noted that, while the *TP53* region is highly rearranged, only 1/23 rearranged samples have been identified as chromothriptic in that region compared with 5/9 samples with *ATRX* rearrangements (Fisher's exact test, $p < 0.01$, odds ratio = 23.5, Figure S2G). These data suggest that chromothripsis is an infrequent mechanism of *TP53* disruption in USARCs and that inactivation of tumor suppressor genes rather than activation of oncogenes is the sine qua non of the USARC rearrangement phenotype. A gene-agnostic method was then used to widen our search for recurrently rearranged regions. Three genomic windows incorporating 5p15.33, 17p13.1, and 17p11.2 were identified as harboring significant rearrangements across samples (false discovery rate [FDR]: $q < 0.2$) (Figure 1F). The 17p13.1 region harbors *TP53*.

Two other recurrently rearranged regions containing canonical cancer driver genes *RB1* and *CDKN2A* were identified by this method but were not significant following multiple testing (FDR: $q \geq 0.2$).

There was an enrichment of diverse rearrangements on the boundaries of the *TERT* gene (5p15.33), which encodes the catalytic subunit of telomerase (Figure S3A). One mechanism of activating *TERT* is through rearrangements that colocalize *TERT* with distant enhancer regions, so-called "enhancer hijacking" (Peifer et al., 2015). By overlapping the genomic positions of the boundaries of structural breakpoints with the dbSUPER (Khan and Zhang, 2016) database of 91 human and mouse tissue types, we identified that of the 13 rearrangements (8 translocations, 4 inversions, and 1 tandem-duplication; 7 downstream, 5 upstream, and 1 within TERT) within 100 kb of *TERT*, 8 rearrangements have a partner region that directly overlaps or is within 500 kb of a super enhancer in all tissues or muscle tissues only. Furthermore, in those samples with such rearrangements we found that the expression of *TERT* was significantly increased (Figure S3B). These data strongly suggest that *TERT* enhancer capture is being tagged by rearrangements in the region. Because *TERT* is known to be dysregulated through multiple mechanisms, this finding prompted a search for other potential mechanisms of *TERT* activation. There were no predicted *TERT* fusion events. However, two cases (PD26857a and PD31187a) demonstrated hypermethylation of the repressive element within the *TERT* promoter locus (Figure S3C). We also found evidence for increased telomere length (tumor:normal ratio) in the majority of samples and disruption in either *ATRX* or *DAXX* or in the *TERT* promoter (Figures S3D and S3E).

To determine the significance of rearrangements in the 17p11.2 region we correlated gene expression for all genes in the cytoband with rearrangement status. This revealed two genes with significantly altered expression namely *GID4* and *RASD1* (Figure S3F); *GID4* encodes a coactivator of RNA polymerase II and has increased expression in the rearranged samples ($p = 2.0 \times 10^{-3}$, $q = 5.6 \times 10^{-3}$). *RASD1*, encoding a member of the RAS family, has a significantly reduced expression in rearranged samples ($p = 5.9 \times 10^{-4}$, $q = 2.6 \times 10^{-3}$). In line with this observation, it has previously been proposed that *RASD1* is a tumor suppressor gene in some cancer types (Gao et al., 2017).

Integration of Driver Mutations, Rearrangements, and Copy-Number Variants

Using a statistical model for mutational driver analysis we found four recurrent driver genes from WGS, all of which are known cancer genes (*TP53*, *RB1*, *PTEN*, and *ATRX*, $q < 0.2$; Table S2). In addition, using this method *MEN1* was identified as somatically mutated in four samples (6%). *MEN1* mutations have not previously been reported in sarcomas and have been described only occasionally in benign smooth muscle tumors and rarely in lipomas (Forbes et al., 2017). These mutations included two frameshift deletions (PD26863a p.F370Sfs*65; PD26873a p.R521Gfs*43), a nonsense mutation (PD31196a p.R532*) and an in-frame deletion (PD26877a p.G168_L173delinsV). Copy-number calling (Table S2) followed by recurrent copy-number analysis revealed significant recurrent altered regions of the USARC genome including amplification of the known sarcoma driver oncogenes *JUN* and *RICTOR*, and deletion of cancer driver genes such as *TP53*, *RB1*, *CDKN2A*, *CBFA2T3*, *STK11*, *TCF3*, and *CYLD* (GISTIC, $q < 0.1$; Figure 2A; Table S2).

We then integrated mutational and structural variants into a comprehensive USARC driver mutation landscape (Figure 2B),

(B) SNV and indel mutational burden barplot (top) and copy-number alterations, SNVs, small indels, structural variants and promoter methylation alterations in known cancer genes (middle), with clinical and genetic covariates (bottom). Red text indicates driver genes identified by dNdSCV ($q < 0.2$). stab, genome stability; CC, cell cycle; tel, telomere maintenance; mTOR, mTOR signaling pathway; men, MENIN pathway; repair, DNA repair. Samples are ordered by sequencing platform, burden group, and mutational status.
See also Table S2.

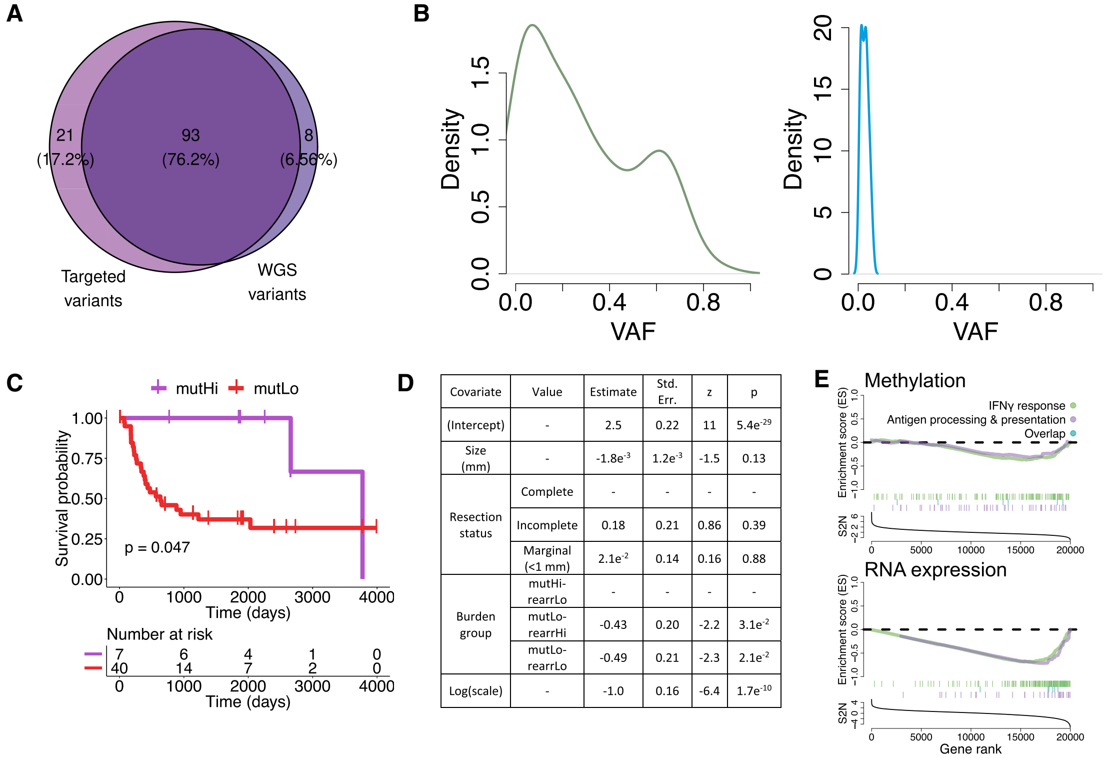

D

Covariate	Value	Estimate	Std. Err.	z	p
(Intercept)	-	2.5	0.22	11	$5.4e^{-29}$
Size (mm)	-	$-1.8e^{-3}$	$1.2e^{-3}$	-1.5	0.13
Resection status	Complete	-	-	-	-
	Incomplete	0.18	0.21	0.86	0.39
	Marginal (<1 mm)	$2.1e^{-2}$	0.14	0.16	0.88
Burden group	mutHi-rearrLo	-	-	-	-
	mutLo-rearrHi	-0.43	0.20	-2.2	$3.1e^{-2}$
	mutLo-rearrLo	-0.49	0.21	-2.3	$2.1e^{-2}$
Log(scale)	-	-1.0	0.16	-6.4	$1.7e^{-10}$

Figure 3. Implications of Increased Tumor Mutational Burden

(A) Venn diagram of predicted pathogenic variants from mutHi samples identified from targeted sequencing (left) and identified from WGS in regions overlapping the design of the targeted baitset (right).

(B) Variant allele frequency (VAF) of all variants (left) or variants only observed by targeted sequencing (right).

(C) Overall survival of patients stratified by mutational burden and with a univariate Kaplan-Meier model.

(D) Multivariate accelerated failure time model for progression-free survival with size of tumor, resection status, and burden group as covariates.

(E) Gene set enrichment analysis for interferon gamma response (green) and antigen presentation (purple) pathways using both DNA methylation (top) and gene expression (bottom) data comparing the mutHi-rearrLo group against all others. See also Table S3.

noting from the WGS data that up to 50% of driver events in *TP53*, *RB1*, and *ATRX* would have been missed if only exome data were available for the cohort. Furthermore, from a potential therapeutic standpoint, mutational profiling and manual curation of driver variants revealed that 33% (n = 17) of tumors harbored mutations in genes encoding proteins upstream of mammalian target of rapamycin (mTOR) in the signaling cascade. These included truncating events in *PTEN* (n = 10), *TSC1* (n = 2), and *TSC2* (n = 2), as well as a hotspot mutation in *PIK3CA* (p.H1047R).

Hypermutation Fuels Subclonal Mutations in Cancer Genes

Data showing that sarcomas have relatively modest mutational burdens compared with other cancer types (Campbell et al., 2017) prompted an investigation to determine whether high-depth sequencing might reveal a wider spectrum of cancer driver genes than identified by our 70× depth for WGS. We therefore sequenced our 7 hypermutated samples and an extension cohort of 16 new USARC samples (Table S3) to a mean target depth of 403× on a cancer driver gene panel. Analysis of the hypermutated samples revealed that up to 18% of variants had not been reported in the WGS (Figure 3A) and were dominated by subclonal mutations (Figure 3B). In the extension cohort, the spectrum of driver genes mirrored that of the WGS data (Figure 2B), which is also reflected in the TCGA.USARC samples. We also found three hypermutated samples (~19%) in the extension cohort, one of which harbored a nonsense mutation of *PALB2* (pE331*). Two samples lacked an identifiable causative mutation in either the somatic or germline genome using the targeted approach.

Utility of a Genomic Classification for USARCs

The histological classification of USARCs has a chequered history and is based on exclusion of other sarcoma entities (Fletcher, 1992). There is a pressing need to address the inconsistencies of the current classification because the morphologically heterogeneous nature of USARCs and its genomic complexity have resulted in a deficiency in identification of biomarkers that could inform risk stratification and clinical management strategies. To test whether classifying USARCs by mutational burden might have prognostic significance we conducted a survival analysis using both a univariate analysis (Figure 3C) and a robust parametric statistical model using multivariate clinical and molecular data including the mutational subgroups (Figure 3D). This revealed that the mutHi group showed significantly better progression-free and metastasis-free survival (Table S3) compared with mutLo groups. As

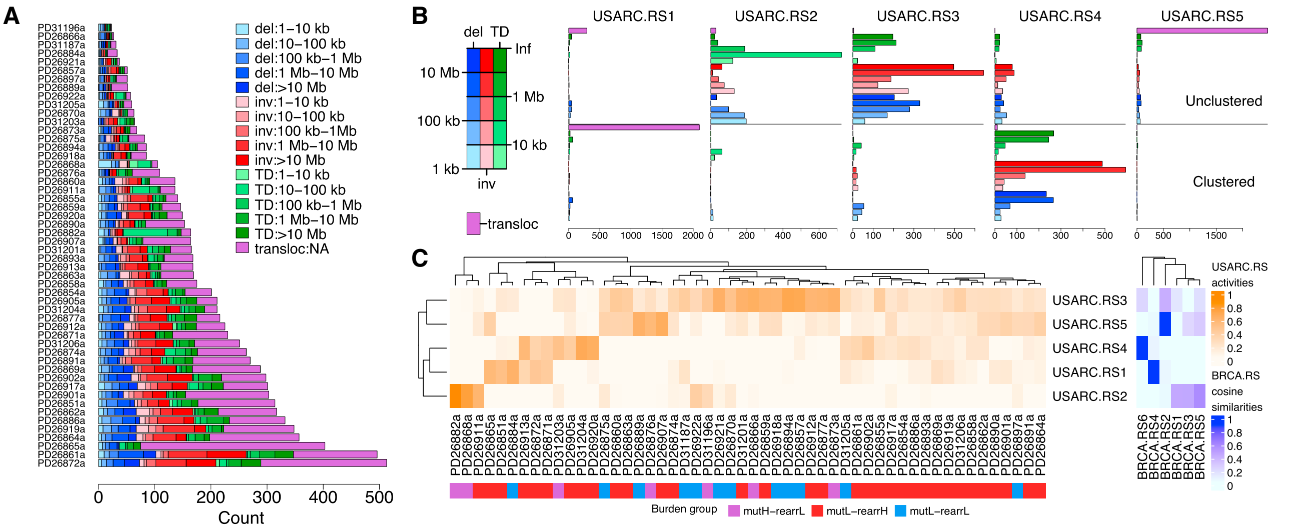

Figure 4. Rearrangement Signatures

(A) Rearrangement diversity and counts in the USARC cohort, classified by rearrangement size and rearrangement class.

(B) Five rearrangement signatures identified by non-negative matrix factorization (NMF); USARC.RS1, clustered translocations (tloc, purple); USARC.RS2, small unclustered tandem duplications (TD, green), inversions (inv, red), and deletions (del, blue); USARC.RS3, large unclustered TDs, invs, and dels; USARC.RS4, large clustered TDs, invs, and dels; USARC.RS5, unclustered tlocs. x axis, strength of each rearrangement class in each signature.

(C) Contribution of activities of each signature per sample (left) and cosine similarities between published breast cancer rearrangement signatures (BRCA.RS1-6) and USARC rearrangement signatures (right). See also Figure S4 and Table S4.

expected, metastasis was found to have a significantly detrimental effect on overall survival (Table S3). There was no significant survival effect associated with any of the most recurrently mutated genes after accounting for clinical confounders. Furthermore, pathway analysis revealed significant enrichment for immune-related pathways in mutHi compared with other samples, suggesting an altered immune response in those samples with a high tumor mutational burden (Figure 3E). The same trend was observed in TCGA hypermutators, although the sample size (n = 3) was not sufficient for a significant result (data not shown).

Rearrangement Signatures Reveal Distinct Patterns of Structural Variation

The rearrHi and rearrLo molecular subgroups highlight the diversity in rearrangement burden in USARCs, and, indeed, the nature of structural rearrangements in USARCs is also varied (Figure 4A). We sought to investigate the rearrangement processes underpinning this landscape. To this end we extracted recurring rearrangement signatures (Nik-Zainal et al., 2016) based on the nature, size distribution, and local clustering of structural variants. Using a non-negative matrix factorization mathematical framework, we identified five predominant signatures (USARC.RS1-USARC.RS5; Figure 4B; Table S4). Interestingly USARC.RS1 and USARC.RS5 were strongly dominated by translocations and showed a comparative dearth of other rearrangement classes. These signatures are differentiated from each other by the presence of proximity clustering of the breakpoint regions, suggestive of alternate rearrangement mechanisms (Glodzik et al., 2017). The remaining signatures (USARC.RS2 to USARC.RS4) showed a more varied pattern of rearrangement classes.

By contrasting these five sarcoma signatures with a published WGS dataset of breast cancer (Nik-Zainal et al., 2016), we found that USARC.RS1, USARC.RS4, and USARC.RS5 have previously been identified, suggesting common mechanisms driving structural variation between these diverse cancer types (Figure 4C). Unsurprisingly, the activities of the two signatures of clustered rearrangements, USARC.RS1 (clustered translocations) and USARC.RS4 (clustered tandem duplications, inversions and deletions), were associated with the number of chromothriptic chromosomes (linear regression, $p = 3.3 \times 10^{-5}$, $q = 8.7 \times 10^{-4}$; $p = 1.1 \times 10^{-2}$, $q = 8.7 \times 10^{-2}$, respectively; Figure S4). USARC.RS2 signature activity was seen in 52% of samples (median exposure = 7%, interquartile range [IQR] = 0%–13%) and is characterized by unclustered co-occurring megabase scale tandem duplications, deletions, and inversions. USARC.RS3, which was seen in 92% of samples (median exposure = 30%, IQR = 23%–45%), has a similar overall pattern to USARC.RS2, but shows a different segment size distribution favoring longer lengths, possibly indicating divergent mechanisms of generation. This intriguing set of results called for a deeper understanding of the impact of extensive and complex structural variation on the copy-number profiles of USARCs.

The Genomic Complexity of USARCs Is Unraveled Using Copy-Number Signatures

Accurate inference of the nature and role of copy-number aberrations in most samples was hindered by substantial complexity, demonstrated by multiple large and small chromosomal gains and losses across the genome and compounded by whole genome duplication (WGD) events. We adopted a pragmatic approach that deconvoluted complex copy-number profiles into various distinct operative copy-number processes and

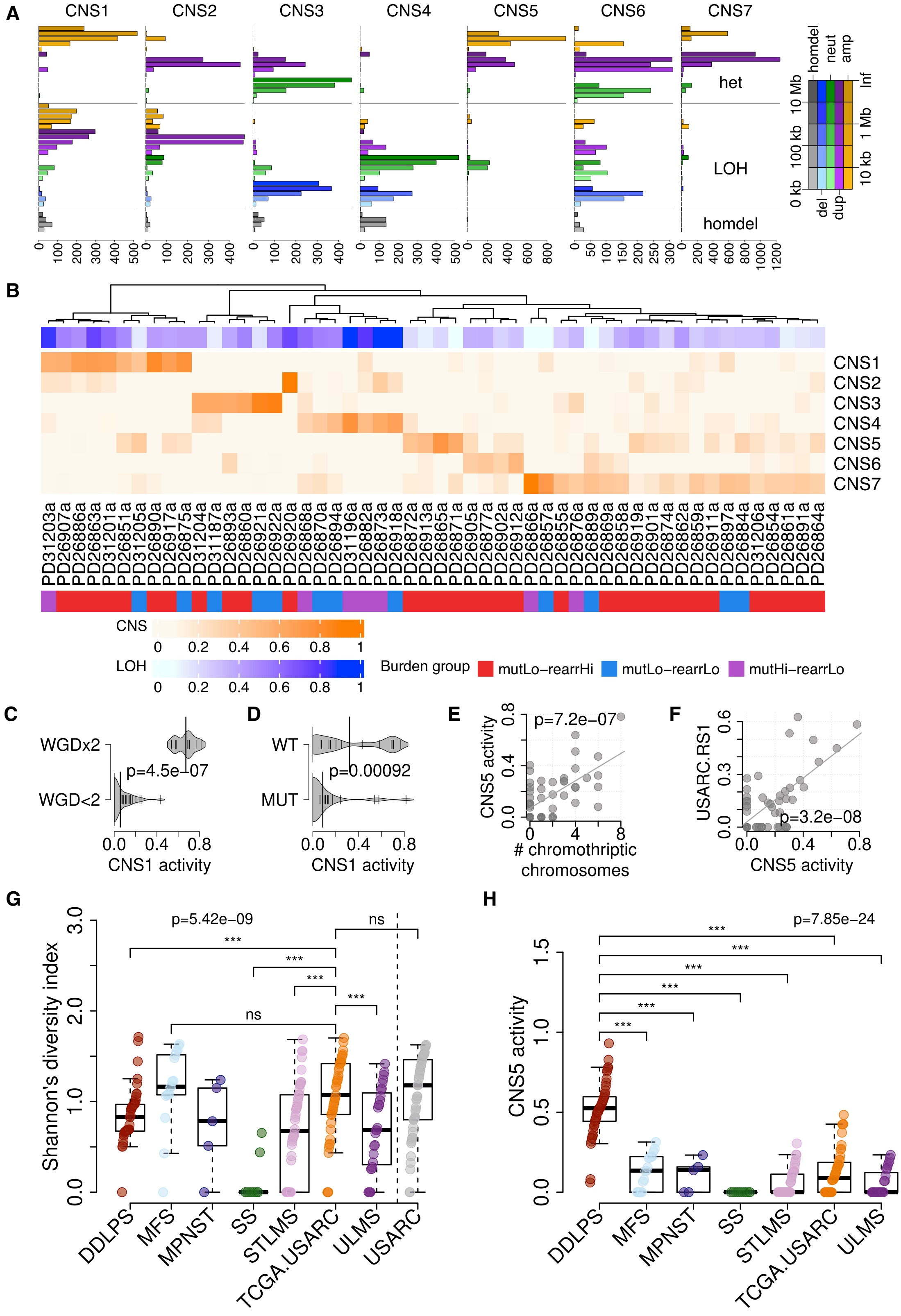

Figure 5. Copy-Number Signatures

(A) Seven copy-number signatures identified using NMF; amp, amplified (CN $\geq$ 1, orange); dup, duplicated (3 $\leq$ CN $\leq$ 4, purple); neut, neutral (CN, 2, green); del, deletion (CN $\leq$ 1, blue); homdel, homozygous deletion (CN, 0, gray); het, heterozygous. x axis, strength of each copy-number class in each signature.

(legend continued on next page)

then compared these across samples to make inferences about their development and effects. Copy-number profiles were summarized into a metric by classifying copy-number segments according to size, LOH status, and total copy number. This framework identified seven copy-number signatures (CNS1-7; Figures 5A and 5B; Table S5). CNS1 is a signature indicative of amplified LOH, which correlates with two or more WGD events (asymptotic Wilcoxon-Mann-Whitney test, $Z = -4.84$, $p = 4.5 \times 10^{-7}$, $q = 2.6 \times 10^{-7}$; Figure 5C). CNS2 is a signature of duplicated LOH that may signify a single WGD. CNS3 is a signature of hypodiploid tumors (no evidence of WGD) with a large proportion of unaltered segments, and some small amplifications and large deletions. CNS4 is a signature of copy neutral LOH. CNS5 has features of amplification with retention of heterozygosity and neutral LOH segments. CNS6 is a complex copy-number signature comprising large heterozygous neutral and duplicated segments with smaller LOH segments of multiple copy-number states. CNS7 is the signature that is observed in the highest proportion of samples (67% of samples) and is a signature of a single WGD. These features of WGD with retention of heterozygosity and losses seen in CNS6 and CNS7 appear to fit a described model of tetraploidization followed by genomic losses, thereby generating an aneuploid cell state, particularly in a p53- and/or RB1-deficient background (Davoli and de Lange, 2012).

Furthermore, our earlier integrative mutational analysis revealed that 68% of tumors harbored a putative disruptive event in *TP53*, and that abrogation of *TP53* was significantly associated with activity of CNS1 (LOH with two or more WGDs) (Kruskal-Wallis test, $p = 9.2 \times 10^{-4}$, $q = 1.3 \times 10^{-2}$; Figure 5D). *PTEN* mutations are associated with an increased activity of the signature of copy neutral LOH, CNS4 (Kruskal-Wallis test, $p = 3.9 \times 10^{-3}$, $q = 4.3 \times 10^{-2}$), and activity of CNS4 is also correlated with tumor mutational burden (linear regression, $p = 5.3 \times 10^{-3}$, $q = 5.1 \times 10^{-2}$). The number of chromothriptic chromosomes in a sample is significantly correlated with activity of CNS5 (linear regression, $p = 7.2 \times 10^{-7}$, $q = 2.6 \times 10^{-5}$; Figure 5E) and samples that harbor chromothriptic chromosomes are enriched in the rearrHi group but are not associated with metastasis (Figures S5A and S5B). Activity of CNS5 across the cohort is also highly similar to the activity of the rearrangement signature of clustered translocations, USARC-RS1 (cosine similarity = 0.82; linear regression, $p = 3.2 \times 10^{-8}$, $q = 5.8 \times 10^{-6}$; Figure 5F). We then sought to determine the effects of these intriguing copy-number patterns on gene expression. We found a consistent and strong gene dose effect of the DNA copy number on RNA expression (Figure S5C); however, there was no consistent pathway enrichment correlated with copy-number groups, compatible with the fact that most of the copy-number events are private events. Further, linear modeling of gene expression in genes of interest identified a significant relationship between gene expression and copy number in five genes (*TP53*, *RB1*, *CDKN2A*, *PTEN*, and *TERT*; $q < 0.05$), but no significant association between promoter methylation and gene expression once copy number is accounted for ($q > 0.05$).

USARCs Show Extreme Copy-Number Heterogeneity

To explore the extent to which the copy-number signatures were operative in other samples, we compared our findings with a cohort of 320 sarcomas of multiple subtypes including samples from TCGA, the allele-specific copy-number states of which were extracted from WGS and high-resolution SNP arrays. Three signatures were identified in this validation cohort (Figures S6A–S6D), all three of which were also identified in USARCs (CNS1, CNS3, and CNS7). CNS3, which is a signature of hypodiploid tumors, was highly operative in synovial sarcoma and a spectrum of low-grade sarcomas, both of which have low karyotypic complexity and rarely show WGD.

We then quantified the within-sample copy-number heterogeneity among different sarcoma types using a diversity index. This demonstrated that the lowest copy-number diversity is seen in synovial sarcoma, which is typically dominated by CNS3. The two USARC cohorts have the same degree of copy-number diversity, being the highest among various sarcoma types and is indistinguishable from the copy-number diversity seen in myxofibrosarcoma (MFS) (Figure 5G). This indicates that multiple processes generate copy-number alterations in USARCs, leading to a highly chaotic and varied copy-number landscape within each sample and across samples. Interestingly, dedifferentiated liposarcoma (DDLPS), a high-grade sarcoma that can show similar morphological heterogeneity and pleomorphism to USARCs showed a significantly lower CNS diversity (Mann-Whitney test; $p = 0.003$). This reduced diversity in DDLPS is reflected by a predominance of CNS5 in this tumor type (Figure 5H). Chromothripsis, particularly of chromosome 12, is a key driver event in DDLPS pathogenesis (Garsed et al., 2014), which is identified in these samples through the observation of CNS5. These findings illustrate that DDLPS samples have more similar characteristics to each other than USARC samples do.

Pseudohaploidization Is Recurrent in USARCs

In view of the large proportion of our cohort of USARC samples bearing different spectra of LOH and the identification of copy-number signatures strongly defined by an LOH pattern, e.g., CNS4, we investigated this phenomenon in greater detail. We found that 14 tumors (27%) in our USARC cohort demonstrate widespread LOH (>50% genome LOH) and that 3 tumors exhibit striking near-genome-scale haploidy (>90% genome LOH).

(B) Activities of copy-number signatures (CNS) per sample, with associated proportion of the genome that shows LOH, and molecular classification groups.
(C) Density plot of CNS1 activity stratified by whether the sample has one or fewer genome doubling events (WGD<2) or has two genome doubling events (WGD×2). Thickness of gray region indicates density. Small vertical lines, data points. Large vertical lines, median.
(D) Density plot of CNS1 activity stratified by *TP53* mutation status.
(E) Scatterplot of CNS5 activity against number of chromothriptic chromosomes. Gray line indicates linear fit.
(F) Scatterplot of CNS5 activity against USARC.RS1 activity. Gray line indicates linear fit.
(G) Diversity estimates of CNS in TCGA sarcoma subtypes and our USARC cohort.
(H) CNS5 activity stratified by tumor type in TCGA.
Boxes show lower quartile, median, and upper quartile; lines denote furthest point within 1.5× the interquartile range away from the box. See also Figures S5 and S6 and Table S5.

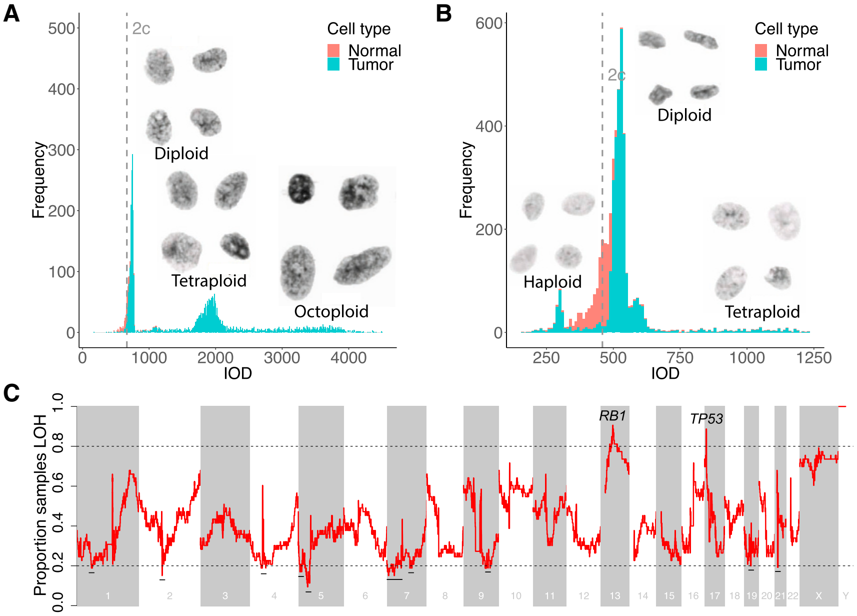

Figure 6. LOH and Haploidization Are Frequent Events in USARCs

(A) Histogram of DNA content, measured as integrated optical density (IOD) (x axis), for cell nuclei from PD26890. Proportion of genome LOH = 44%. 2c, median IOD of normal cell nuclei.

(B) Histogram of DNA content for cell nuclei from PD26873. Proportion of genome LOH = 93%.

(C) Proportion of samples within the USARC WGS cohort that are LOH (y axis) in sliding windows of the human genome of size 1 Mb each separated by 100 kb (x axis). Dashed line, boundary of regions with highly recurrent LOH (>0.8) or retention of heterozygosity (<0.2). Regions with retention of heterozygosity are highlighted with a horizontal black line. Regions with recurrent LOH are labeled with putative driver tumor suppressor genes in those regions.

These data suggest that haploidization may be a common event in USARC evolution. To independently investigate this mechanism, we carried out ploidy analysis to estimate both DNA content and visualize nuclear morphology. This revealed diverse cell states with multiple cell fractions of increasing ploidy indicative of successive WGDs in samples (Figure 6A). One sample revealed an intact near-haploid subclone that constituted 9% of tumor cells, suggesting that the genome-duplicated clone had not completely swept through the tumor in this sample and might have been fueled by the near-haploid population (Figure 6B). Large-scale haploidy has previously been described using SNP arrays in low-grade chondrosarcoma (Bovee et al., 2000) and in other sarcoma subtypes (Mertens et al., 1998) of various grades.

Recurrent regions of LOH included the loci of *RB1* (chromosome 13q14.2) and *TP53* (chromosome 17p13.1) (Figure 6C), with 88% and 63% of mutational events co-occurring with LOH, respectively (Figure 2B), highlighting how the LOH background makes USARCs propitious to double-hits on tumor suppressors. Conversely, there were retained regions of heterozygosity on chromosomes 1, 2, 4, 5, 7, 9, 19, and 21 in >80% of samples (Figure 6C). Proliferation rates determined by mitotic counts did not differ between samples with and without LOH and aberrant mitoses were prevalent in all samples (data not shown). Finally, we investigated the relationship between LOH and chromothripsis across our dataset and found negative associations between CNS5, the signature of chromothripsis, and both the proportion of genome that is LOH (linear regression, $p = 2.1 \times 10^{-4}$, $q = 3.8 \times 10^{-3}$), and the sum of all LOH-associated signatures, CNS1-4 (linear regression, $p = 2.1 \times 10^{-7}$, $q = 2.0 \times 10^{-5}$).

Timing of Polyploidization and Driver Mutations in Sarcomas

To better understand the contribution of polyploidization to the tumorigenesis of USARCs we used the copy-number data to interrogate patterns of WGD. A total of 89% of samples exhibited at least one WGD event, and 19% of samples showed at least two WGDs (Figure 7A). This contrasted remarkably with the average WGD occurrence of 37% across multiple cancer types (Zack et al., 2013), indicating that WGD is an important tumorigenic event in USARCs. Mutational data were then integrated with the WGD analysis to infer both relative and real-time timing of WGD events. Categorizing samples by their most prevalent copy-number signature (Figures 7B–7D) revealed differential WGD timing in USARCs; groups of samples with potentially multiple WGDs, CNS5 and CNS1, have predominantly early first WGD, whereas CNS4 tumors have predominantly late WGD events, with the exception of two of the hypermutators in that group. The molecular timing of second WGDs, in cases where WGD was amenable to timing (Table S6), showed that most occurred close to diagnosis, whereas the first WGD arose across a range of molecular times. The time between the first and second WGD is often in the order of decades, while the time between second WGD and diagnosis is considerably shorter. This analysis was extended to the sarcoma cohort of TCGA (Figure 7E), that showed the largest range of WGD times in USARCs, and confirmed early WGD in CNS5, drastically contrasting the late WGD in DDLPS of the same signature group.

Timing of driver mutations demonstrated that the vast majority of driver mutations occurred before the first WGD in USARCs (Figure 7D). Of interest in the USARC cohort are three samples with age of diagnosis <40 years (within the 5% quantile of diagnostic age of USARCs and MFS; mean = 67.8 years), all of which have a late WGD. Indeed, there is a trend toward late WGD in patients with a younger age at diagnosis (linear regression, $p = 0.066$). Extending this analysis to the sarcoma cohort of TCGA (Figures 7E and S7A–S7F) revealed an enrichment for a second WGD (CNS1) in USARC/MFS. WGD was predominantly late in CNS5 DDLPS, and predominantly early in CNS5 USARC, confirming the results in our cohort. Remarkably, in CNS1, which was associated with *TP53* LOH, the two *TP53* mutations amenable to timing in USARCs seemed to have occurred posterior to the first WGD but before the second WGD, suggesting that while the first WGD in these samples restored the

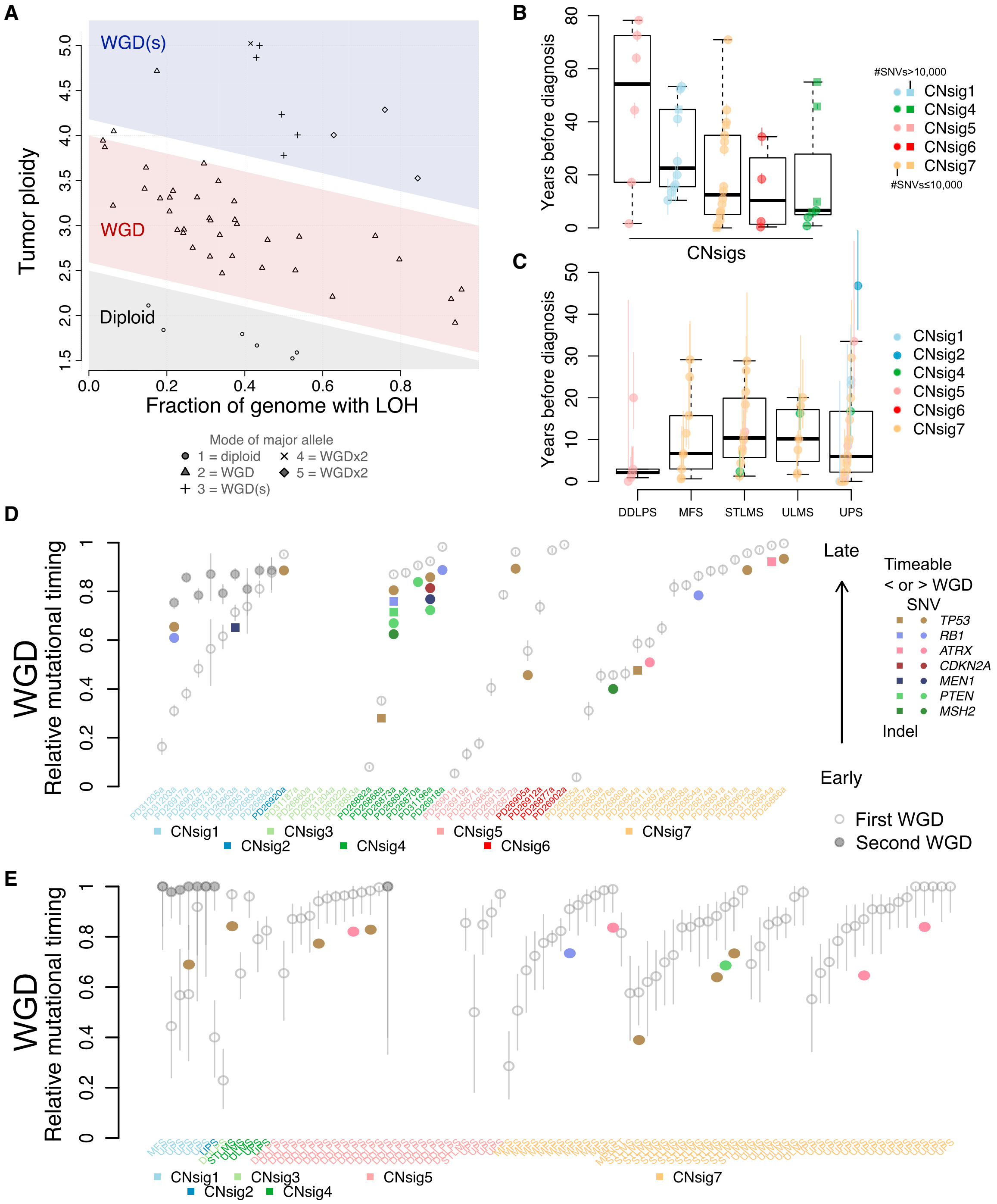

Figure 7. Timing of Genome Duplication and Driver Mutations

(A) The number of WGD determined by the mode of the major allele in a sample (mode 1, diploid, 0×WGD; mode 2, tetraploid, 1×WGD; mode >2, octoploid, 2×WGD) matches inference from the spread of the samples in the proportion of LOH versus ploidy space.

(B) Time of WGD (circles/squares, mean timing per sample. Square indicates more than 10,000 SNVs and circle is less than 10,000 SNVs; vertical colored bars, 95% confidence intervals on the mean values) in years before diagnosis, split by predominant copy-number signature in USARC cohort whole genomes.

(C) Time of WGD in the sarcoma cohort of TCGA, split by tumor type.

(D) Relative timing of driver mutations (colored circles) and WGD events (empty/gray circles) using the mutations as a molecular clock in USARC cohort whole genomes. Vertical bars, 95% confidence intervals. Samples split by predominant copy-number signature.

(legend continued on next page)

diploid copy-number state, a further hit was necessary to reinstate *TP53* inactivation.

USARCs Are Heterogeneous Tumors with Evidence for Subclonal WGD

Our data indicate that polyploidization is a key event in USARC tumorigenesis except for a small outlier group of high-grade tumors without evidence of WGD as estimated by WGS (CNS3). To address this conundrum, we used ploidy cytometry analysis, which revealed that 4/6 ''non-genome duplicated'' CNS3 samples contained substantial cell fractions with one or more WGD events, ranging from 36% to 71% of cells in a sample (Figures 8A and 8B). This discrepancy of ploidy results between WGS and cytometry could be methodological as our WGS algorithm is blind to subclonal WGD, but it also reinforces the heterogeneous nature of these tumors as different regions from the same tumor were used for these two analyses. PD31204 contained large peaks of a relatively diploid clone (44% of cells), and sizable fractions of cells with one or two WGD events (34% and 11% of cells, respectively) (Figure 8B). These results demonstrate sequential subclonal WGD events in samples that have predominantly diploid populations of cells, which suggest that these tumors were diagnosed before the onset of a potential clonal sweep in which the WGD population of cells dominated, and that WGD and second WGD events are probably more prevalent in USARCs than our estimates using WGS of a single tumor region suggest.

DISCUSSION

Through the parallel analyses of WGS, DNA methylation profiling and gene expression we have generated a comprehensive molecular and clinical landscape of USARCs. USARCs with high mutational burden (mutHi) were found to be enriched for activation of immune pathways at both the DNA methylation and gene expression levels. More effective therapeutic approaches are desperately needed for USARC patients and we propose that classifying USARCs by mutational burden is clinically relevant. Furthermore, hypermutation and the recurrence of mutations in mTOR signaling genes open up alternative avenues for stratification and immunotherapy clinical trial design for these patients.

We have illuminated the karyotypic complexity in USARCs through the development of a copy-number signature framework that has proved to be a practical method to infer evolutionary dynamics at a structural level. By integrating the results from copy-number signatures, mutational timing, and ploidy analysis we deduced four potential routes to USARC tumorigenesis, all beginning with early driver mutations, preceding any WGD event (Figure 8C). In particular, CNS4 is indicative of WGD in an inferred precursor cell that has a near-haploid chromosomal state. This genomic loss is likely achieved through a single mis-segregation event such as a mitotic error rather than through progressive loss. Live cell-imaging experiments of chromosomally unstable cells have demonstrated that mis-segregation during anaphase can lead to two aberrant daughter cells; one that is hypoploid, and another that is hyperploid due to sequestration of a lagging chromosome in a micronucleus, which may be susceptible to chromothripsis (Huang et al., 2012). This suggests that there could be a dichotomous relationship between cells that are hypoploid (genome-wide LOH) and those in which chromothripsis can occur. Indeed, USARC samples with large-scale LOH show a negative association with signatures of chromothripsis, suggesting a different evolutionary trajectory between LOH and chromothriptic samples. Alternatively, chromothripsis may be selected against in near-haploid cells due to the potential introduction of large regions of homozygous deletions. The strong association of CNS5 with USARC.RS1, but not USARC.RS4, demonstrates that in USARC chromothripsis most often generates translocations, and suggests that there may be other signatures of chromothripsis that remain to be discovered. On a permissive background of *TP53* or *RB1* inactivation, such widespread genomic loss could lead to a precursor cell state with a near-haploid genome as seen, for example, in samples PD31196a and PD26920a. These losses likely act as a second hit in a genome-wide fashion, unmasking multiple potent pathogenic somatic or recessive germline variants simultaneously, thus dramatically increasing the fitness of an incipient cancer cell in one crisis event. Subsequent WGD through telomere-crisis-induced endoreduplication potentially increases the evolutionary space within which such a cell can optimize the dosage of various genes without risking further haploidy or loss of any survival-critical genes (Davoli and de Lange, 2012), and, in fact, we observed gene-dosage effects dependent on copy number through our integrative analysis. Conversely there is no significant association between methylation status of driver genes and gene expression once copy number is accounted for. Furthermore, WGD dramatically accelerates tumor development leading to diagnosis soon after WGD in some cases of USARCs. In other cases, the first WGD event ranges from just before diagnosis to multiple decades before diagnosis, suggesting that other factors contribute to rapid tumorigenesis in some tumors but not in others. In contrast, a second WGD event is consistently late in the tumor's history, sometimes decades after the first duplication and occurring just before diagnosis. This long latency provides a clinically relevant time frame for intervention should the early driver mutations be amenable to detection in the blood circulation. Key features from the sequencing data corroborated by DNA ploidy experiments, mutational timing, and cytogenetic findings expand the view that WGD is a recurrent phenomenon in USARCs and is a potential transformation event instrumental in their development.

These new models of sarcoma development demonstrate likely punctuated evolutionary trajectories and provide insights into how patterns of LOH and copy-number gain sculpt the sarcoma genome. Future work on larger cohorts collected prospectively may elucidate other mechanisms underpinning the aberrant copy-number landscape in sarcomas and may yield further undiscovered copy-number signatures. There is also a

(E) Relative timing of driver mutations and WGD events in the sarcoma cohort of TCGA. Samples split by predominant copy-number signature, and subdivided by tumor type. Vertical bars, 95% confidence intervals.

Boxes are delimited by first and third quartiles; the thick segment shows the median; and whiskers extend to the last data points within 1.5 of the box length away from the box. See also Figure S7 and Table S6.

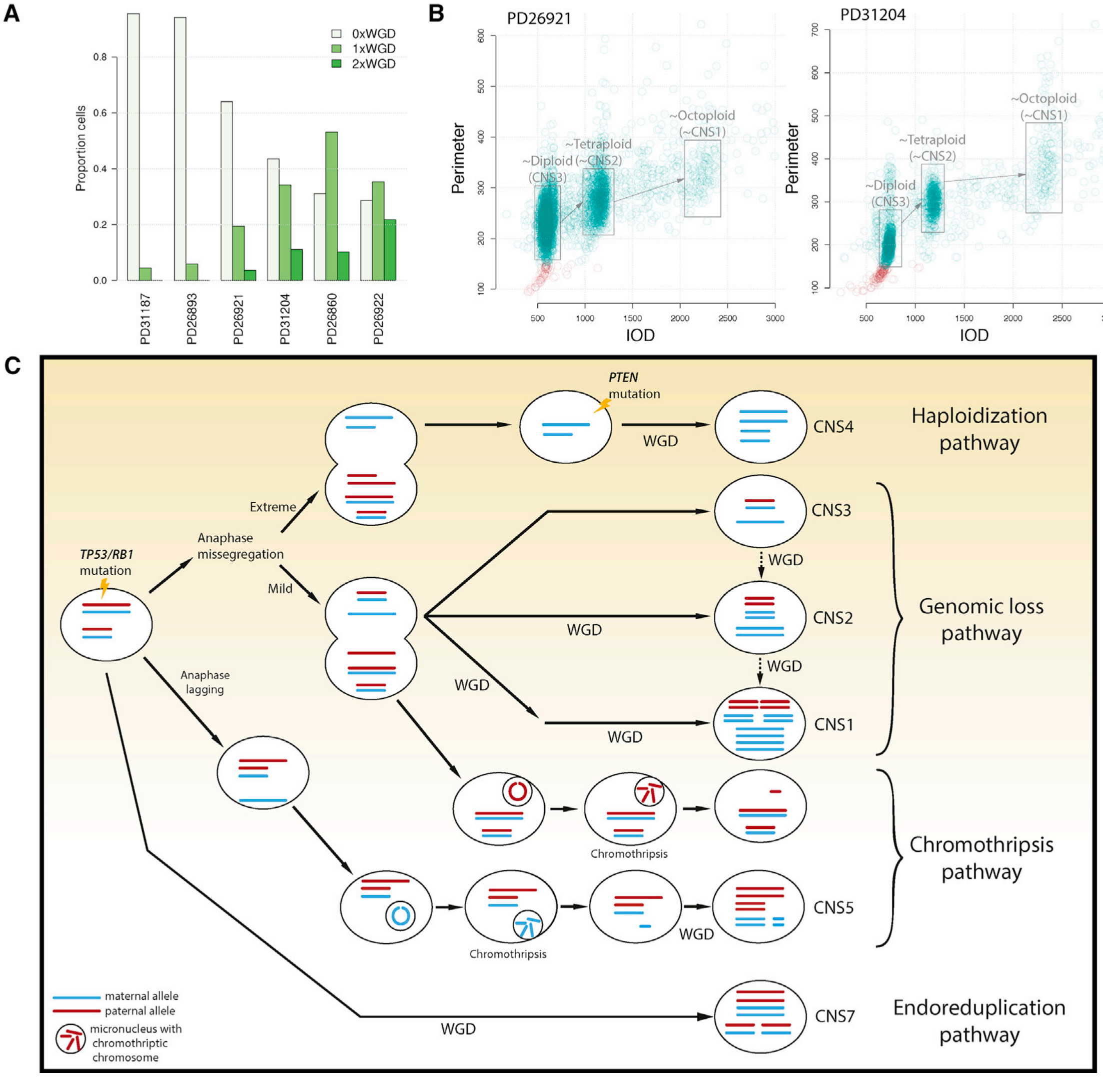

Figure 8. Evolutionary Pathways in USARCs

(A) Proportion of cells within a sample with no WGD (0×WGD), one WGD (1×WGD), or two WGDs (2×WGD) using cytometric ploidy analysis, for six samples estimated to be non-WGD through WGS.

(B) Representative examples of ploidy results for CNS3 (diploid) samples. Ploidy displayed as integrated optical density (x axis) and nuclear perimeter (y axis) of each nucleus.

(C) Proposed pathways of USARC tumorigenesis. Driver mutations (*TP53* and *RB1*) are early events in USARCs. Haploidization pathway: extreme anaphase mis-segregation associated with near-genome-wide haploidy, which is rescued by WGD, leading to a CNS4 pattern. Genomic loss pathway: less extreme anaphase mis-segregation generates large areas of LOH. Three signatures (CNS3, CNS2, and CNS1) that are variations of this LOH pattern but differentiated from each other by subsequent WGD. Chromothripsis pathway: anaphase mis-segregation or anaphase lagging could also lead to chromosomal micronucleation. CNS5 is a signature of this process followed by WGD. Endoreduplication pathway: a tumor cell may undergo WGD with relatively few other copy-number alterations: CNS7.

requirement to refine these models by investigating karyotypic instability at multi-region and single-cell resolution to understand better the operative dynamics of tumor progression through copy-number evolution.

ETHICS

Informed consent was obtained from all subjects and ethical approval for samples obtained from Cambridgeshire 2 Research Ethics Service (reference: 09/H0308/165). Approval to conduct the study was granted by NHS Health Research Authority (REC reference: 16/NW/0769).

STAR★METHODS

Detailed methods are provided in the online version of this paper and include the following:

- KEY RESOURCES TABLE
- CONTACT FOR REAGENT AND RESOURCE SHARING

- EXPERIMENTAL MODEL AND SUBJECT DETAILS
  - Patient Samples
  - Case Selection
- METHOD DETAILS
  - Tissue Processing and DNA Extraction
  - Whole-Genome Sequencing Protocol and Data Processing
  - Targeted Sequencing Protocol and Data Processing
  - RNA Sequencing Protocol and Data Processing
  - Methylation Protocol
  - Immunohistochemistry Protocol
  - Fluorescence *In-Situ* Hybridization Protocol
  - Image Cytometry Protocol
- QUANTIFICATION AND STATISTICAL ANALYSIS
  - Somatic Mutation Triaging
  - Germline Mutation Triaging
  - Rearrangement Triaging
  - Copy-Number Calling
  - Recurrent Rearrangements
  - Recurrent Copy Number Alterations
  - Chromothripsis Identification
  - Telomere Length Estimation
  - Mutational Signatures
  - Rearrangement Signatures
  - Copy Number Signatures
  - Validation Cohort
  - Survival Analysis
  - Timing of Whole Genome Duplications
  - Real-Time Timing
  - Gene Set Enrichment Analysis (GSEA)
  - Linear Modelling of Gene Expression
  - Statistical Analysis
- DATA AND SOFTWARE AVAILABILITY
  - Software Availability
  - Data Availability

SUPPLEMENTAL INFORMATION

Supplemental Information includes seven figures and six tables and can be found with this article online at https://doi.org/10.1016/j.ccell.2019.02.002.

ACKNOWLEDGMENTS

Samples were obtained from the RNOH Musculoskeletal Research Program and Biobank with technical support provided by staff members Miss Maia Rocha, Mr Lohkesh Ramsurn, Miss Bhavisha Khatri, and Miss Rebecca Gibbons. UCL Genomics – methylation arrays. UCL Pathology Core facility and UCL Advanced Diagnostics staff – technical support. CRUK-UCL Center sequencing core facility – RNA sequencing. Funding was provided to A.M.F. and S.J.S. by the NIH Research, UCLH Biomedical Research Centre, and to A.M.F. by the CRUK UCL Experimental Cancer Center. A.F. receives funding from the BBSRC (BB/R009295/1), PCUK (MA-TR15-009), and Medical Research Council (MR/M025411/1). P.V.L. is a Winton Group Leader in recognition of the Winton Charitable Foundation's support toward the establishment of the Francis Crick Institute. S.B. is personally funded through a Wellcome Trust Intermediate Clinical Research Fellowship, P.J.C. through a Wellcome Trust Senior Clinical Research Fellowship, M.J. and N.P. through CRUK Clinician Scientist Fellowships. C.D.S. receives funding through CRUK. M.T. is a postdoctoral fellow supported by the European Union's Horizon 2020 research and innovation program (Marie Skłodowska-Curie Grant agreement no.747852-SIOMICS). This work was supported by the Francis Crick Institute, which receives its core funding from Cancer Research UK (FC001202), the UK Medical Research Council (FC001202), and the Wellcome Trust (FC001202). Part of the results published here are based on data generated by TCGA Research Network: http://cancergenome.nih.gov. We are grateful to the patients for participating in this research and to the clinician teams involved in their care.

AUTHOR CONTRIBUTIONS

Conceptualization, N.P., P.J.C., and A.M.F.; Formal Analysis, C.D.S., M.T., A.P.W., P.L., and N.P.; Software, C.D.S., M.T., S.B., G.C., P.L., and K.H.; Mutational Signatures, L.B.A.; Image Cytometry, C.D.S. and D.O.; Driver Analysis, C.D.S., P.S.T., and I.M.; Clinical Data, S.J.S., A.M.F., N.P., R.T., and F.A.; Immunohistochemistry and FISH, F.B., H.Y., N.P., and M.J.; RNA Sequencing, P.D. and H.V.; Sample Curation, R.T., F.A., A.M.F., and N.P.; Data Curation, N.P. and C.D.S.; Writing – Original Draft, Review & Editing, C.D.S., M.T., P.V.L., A.M.F., and N.P.; Supervision, N.P., P.V.L., and A.F.

DECLARATIONS OF INTERESTS

The authors declare no competing interests.

Received: June 15, 2018
Revised: November 12, 2018
Accepted: February 6, 2019
Published: March 18, 2019

REFERENCES

AACR Project GENIE Consortium (2017). AACR project GENIE: powering precision medicine through an international consortium. Cancer Discov. *7*, 818–831.

Alexandrov, L., Kim, J., Haradhvala, N.J., Huang, M.N., Ng, A.W.T., Boot, A., Covington, K.R., Gordenin, D.A., Bergstrom, E., Lopez-Bigas, N., et al. (2018). The repertoire of mutational signatures in human cancer. bioRxiv. https://doi.org/10.1101/322859.

Aryee, M.J., Jaffe, A.E., Corrada-Bravo, H., Ladd-Acosta, C., Feinberg, A.P., Hansen, K.D., and Irizarry, R.A. (2014). Minfi: a flexible and comprehensive bioconductor package for the analysis of Infinium DNA methylation microarrays. Bioinformatics *30*, 1363–1369.

Van der Auwera, G.A., Carneiro, M.O., Hartl, C., Poplin, R., Del Angel, G., Levy-Moonshine, A., Jordan, T., Shakir, K., Roazen, D., Thibault, J., et al. (2013). From FastQ data to high confidence variant calls: the Genome Analysis Toolkit best practices pipeline. Curr. Protoc. Bioinformatics *43*, 11.10.1–33.

Behjati, S., Tarpey, P.S., Haase, K., Ye, H., Young, M.D., Alexandrov, L.B., Farndon, S.J., Collord, G., Wedge, D.C., Martincorena, I., et al. (2017). Recurrent mutation of IGF signalling genes and distinct patterns of genomic rearrangement in osteosarcoma. Nat. Commun. *8*, 15936.

Bellacosa, A., Cicchillitti, L., Schepis, F., Riccio, A., Yeung, A.T., Matsumoto, Y., Golemis, E.A., Genuardi, M., and Neri, G. (1999). MED1, a novel human methyl-CpG-binding endonuclease, interacts with DNA mismatch repair protein MLH1. Proc. Natl. Acad. Sci. U S A *96*, 3969–3974.

Bignell, G.R., Greenman, C.D., Davies, H., Butler, A.P., Edkins, S., Andrews, J.M., Buck, G., Chen, L., Beare, D., Latimer, C., et al. (2010). Signatures of mutation and selection in the cancer genome. Nature *463*, 893–898.

Bovee, J.V., van Royen, M., Bardoel, A.F., Rosenberg, C., Cornelisse, C.J., Cleton-Jansen, A.M., and Hogendoorn, P.C. (2000). Near-haploidy and subsequent polyploidization characterize the progression of peripheral chondrosarcoma. Am. J. Pathol. *157*, 1587–1595.

Burrell, R.A., McGranahan, N., Bartek, J., and Swanton, C. (2013). The causes and consequences of genetic heterogeneity in cancer evolution. Nature *501*, 338–345.

Campbell, B.B., Light, N., Fabrizio, D., Zatzman, M., Fuligni, F., de Borja, R., Davidson, S., Edwards, M., Elvin, J.A., Hodel, K.P., et al. (2017). Comprehensive analysis of hypermutation in human cancer. Cell *171*, 1042–1056.e10.

Chang, M.T., Asthana, S., Gao, S.P., Lee, B.H., Chapman, J.S., Kandoth, C., Gao, J., Socci, N.D., Solit, D.B., Olshen, A.B., et al. (2016). Identifying recurrent mutations in cancer reveals widespread lineage diversity and mutational specificity. Nat. Biotechnol. *34*, 155–163.

Davoli, T., and de Lange, T. (2012). Telomere-driven tetraploidization occurs in human cells undergoing crisis and promotes transformation of mouse cells. Cancer Cell *21*, 765–776.

Dentro, S.C., Wedge, D.C., and Van Loo, P. (2017). Principles of reconstructing the subclonal architecture of cancers. Cold Spring Harb. Perspect. Med. *7*, https://doi.org/10.1101/cshperspect.a026625.

Ding, Z., Mangino, M., Aviv, A., Spector, T., and Durbin, R.; UK10K Consortium (2014). Estimating telomere length from whole genome sequence data. Nucleic Acids Res. *42*, e75.

Encode Project Consortium (2012). An integrated encyclopedia of DNA elements in the human genome. Nature *489*, 57–74.

Fletcher, C.D. (1992). Pleomorphic malignant fibrous histiocytoma: fact or fiction? A critical reappraisal based on 159 tumors diagnosed as pleomorphic sarcoma. Am. J. Surg. Pathol. *16*, 213–228.

Fletcher, C.D. (2014). The evolving classification of soft tissue tumours - an update based on the new 2013 WHO classification. Histopathology *64*, 2–11.

Fletcher, C.D., Gustafson, P., Rydholm, A., Willen, H., and Akerman, M. (2001). Clinicopathologic re-evaluation of 100 malignant fibrous histiocytomas: prognostic relevance of subclassification. J. Clin. Oncol. *19*, 3045–3050.

Forbes, S.A., Beare, D., Boutselakis, H., Bamford, S., Bindal, N., Tate, J., Cole, C.G., Ward, S., Dawson, E., Ponting, L., et al. (2017). COSMIC: somatic cancer genetics at high-resolution. Nucleic Acids Res. *45*, D777–D783.

Gao, S., Jin, L., Liu, G., Wang, P., Sun, Z., Cao, Y., Shi, H., Liu, X., Shi, Q., Zhou, X., et al. (2017). Overexpression of RASD1 inhibits glioma cell migration/invasion and inactivates the AKT/mTOR signaling pathway. Sci. Rep. *7*, 3202.

Garsed, D.W., Marshall, O.J., Corbin, V.D., Hsu, A., Di Stefano, L., Schroder, J., Li, J., Feng, Z.P., Kim, B.W., Kowarsky, M., et al. (2014). The architecture and evolution of cancer neochromosomes. Cancer Cell *26*, 653–667.

Gaujoux, R., and Seoighe, C. (2010). A flexible R package for nonnegative matrix factorization. BMC Bioinformatics *11*, 367.

Gerstung, M., Jolly, C., Leshchiner, I., Dentro, S.C., Gonzalez, S., Mitchell, T.J., Rubanova, Y., Anur, P., Rosebrock, D., Yu, K., et al. (2017). The evolutionary history of 2,658 cancers. bioRxiv. https://doi.org/10.1101/161562.

Glodzik, D., Morganella, S., Davies, H., Simpson, P.T., Li, Y., Zou, X., Diez-Perez, J., Staaf, J., Alexandrov, L.B., Smid, M., et al. (2017). A somatic-mutational process recurrently duplicates germline susceptibility loci and tissue-specific super-enhancers in breast cancers. Nat. Genet. *49*, 341–348.

Griffith, M., Spies, N.C., Krysiak, K., McMichael, J.F., Coffman, A.C., Danos, A.M., Ainscough, B.J., Ramirez, C.A., Rieke, D.T., Kujan, L., et al. (2017). CIViC is a community knowledgebase for expert crowdsourcing the clinical interpretation of variants in cancer. Nat. Genet. *49*, 170–174.

Hedley, D.W., Friedlander, M.L., Taylor, I.W., Rugg, C.A., and Musgrove, E.A. (1983). Method for analysis of cellular DNA content of paraffin-embedded pathological material using flow cytometry. J. Histochem. Cytochem. *31*, 1333–1335.

Huang, Y., Jiang, L., Yi, Q., Lv, L., Wang, Z., Zhao, X., Zhong, L., Jiang, H., Rasool, S., Hao, Q., et al. (2012). Lagging chromosomes entrapped in micronuclei are not 'lost' by cells. Cell Res. *22*, 932–935.

Jackson, C.H. (2016). flexsurv: a platform for parametric survival modeling in R. J. Stat. Softw. *70*, https://doi.org/10.18637/jss.v070.i08.

Khan, A., and Zhang, X. (2016). dbSUPER: a database of super-enhancers in mouse and human genome. Nucleic Acids Res. *44*, D164–D171.

Kim, D., Landmead, B., and Salzberg, S.L. (2015). HISAT: a fast spliced aligner with low memory requirements. Nat. Methods *12*, 357–U121.

Koelsche, C., Renner, M., Johann, P., Leiss, I., Sahm, F., Schimmack, S., Wardelmann, E., Renker, E.K., Schirmacher, P., Korshunov, A., et al. (2016). Differential nuclear ATRX expression in sarcomas. Histopathology *68*, 738–745.

Korbel, J.O., and Campbell, P.J. (2013). Criteria for inference of chromothripsis in cancer genomes. Cell *152*, 1226–1236.

Layer, R.M., Chiang, C., Quinlan, A.R., and Hall, I.M. (2014). LUMPY: a probabilistic framework for structural variant discovery. Genome Biol. *15*, R84.

Lek, M., Karczewski, K.J., Minikel, E.V., Samocha, K.E., Banks, E., Fennell, T., O'Donnell-Luria, A.H., Ware, J.S., Hill, A.J., Cummings, B.B., et al. (2016). Analysis of protein-coding genetic variation in 60,706 humans. Nature *536*, 285–291.

Linch, M., Miah, A.B., Thway, K., Judson, I.R., and Benson, C. (2014). Systemic treatment of soft-tissue sarcoma-gold standard and novel therapies. Nat. Rev. Clin. Oncol. *11*, 187–202.

Van Loo, P., Nordgard, S.H., Lingjaerde, O.C., Russnes, H.G., Rye, I.H., Sun, W., Weigman, V.J., Marynen, P., Zetterberg, A., Naume, B., et al. (2010). Allele-specific copy number analysis of tumors. Proc. Natl. Acad. Sci. U S A *107*, 16910–16915.

Martincorena, I., Raine, K.M., Gerstung, M., Dawson, K.J., Haase, K., Van Loo, P., Davies, H., Stratton, M.R., and Campbell, P.J. (2017). Universal patterns of selection in cancer and somatic tissues. Cell *171*, 1029–1041.e21.

McLaren, W., Gil, L., Hunt, S.E., Riat, H.S., Ritchie, G.R., Thormann, A., Flicek, P., and Cunningham, F. (2016). The ensembl variant effect predictor. Genome Biol. *17*, 122.

Mermel, C.H., Schumacher, S.E., Hill, B., Meyerson, M.L., Beroukhim, R., and Getz, G. (2011). GISTIC2.0 facilitates sensitive and confident localization of the targets of focal somatic copy-number alteration in human cancers. Genome Biol. *12*, R41.

Mertens, F., Fletcher, C.D., Dal Cin, P., De Wever, I., Mandahl, N., Mitelman, F., Rosai, J., Rydholm, A., Sciot, R., Tallini, G., et al. (1998). Cytogenetic analysis of 46 pleomorphic soft tissue sarcomas and correlation with morphologic and clinical features: a report of the CHAMP study group. Genes Chromosomes Cancer *22*, 16–25.

Nik-Zainal, S., Davies, H., Staaf, J., Ramakrishna, M., Glodzik, D., Zou, X., Martincorena, I., Alexandrov, L.B., Martin, S., Wedge, D.C., et al. (2016). Landscape of somatic mutations in 560 breast cancer whole-genome sequences. Nature *534*, 47–54.

Peifer, M., Hertwig, F., Roels, F., Dreidax, D., Gartlgruber, M., Menon, R., Kramer, A., Roncaioli, J.L., Sand, F., Heuckmann, J.M., et al. (2015). Telomerase activation by genomic rearrangements in high-risk neuroblastoma. Nature *526*, 700–704.

Pertea, M., Pertea, G.M., Antonescu, C.M., Chang, T.C., Mendell, J.T., and Salzberg, S.L. (2015). StringTie enables improved reconstruction of a transcriptome from RNA-seq reads. Nat. Biotechnol. *33*, 290–295.

R Core Team (2017). R: A Language and Environment for Statistical Computing (R Foundation for Statistical Computing).

Raine, K.M., Hinton, J., Butler, A.P., Teague, J.W., Davies, H., Tarpey, P., Nik-Zainal, S., and Campbell, P.J. (2015). cgpPindel: identifying somatically acquired insertion and deletion events from paired end sequencing. Curr. Protoc. Bioinformatics *52*, 15.7.1–12.

Rohrich, M., Koelsche, C., Schrimpf, D., Capper, D., Sahm, F., Kratz, A., Reuss, J., Hovestadt, V., Jones, D.T., Bewerunge-Hudler, M., et al. (2016). Methylation-based classification of benign and malignant peripheral nerve sheath tumors. Acta Neuropathol. *131*, 877–887.

Rosenthal, R., McGranahan, N., Herrero, J., Taylor, B.S., and Swanton, C. (2016). DeconstructSigs: delineating mutational processes in single tumors distinguishes DNA repair deficiencies and patterns of carcinoma evolution. Genome Biol. *17*, 31.

Savina, M., Le Cesne, A., Blay, J.Y., Ray-Coquard, I., Mir, O., Toulmonde, M., Cousin, S., Terrier, P., Ranchere-Vince, D., Meeus, P., et al. (2017). Patterns of care and outcomes of patients with METAstatic soft tissue SARComa in a real-life setting: the METASARC observational study. BMC Med. *15*, 78.

Stephens, P.J., Greenman, C.D., Fu, B., Yang, F., Bignell, G.R., Mudie, L.J., Pleasance, E.D., Lau, K.W., Beare, D., Stebbings, L.A., et al. (2011). Massive genomic rearrangement acquired in a single catastrophic event during cancer development. Cell *144*, 27–40.

Subramanian, A., Tamayo, P., Mootha, V.K., Mukherjee, S., Ebert, B.L., Gillette, M.A., Paulovich, A., Pomeroy, S.L., Golub, T.R., Lander, E.S., et al. (2005). Gene set enrichment analysis: a knowledge-based approach for interpreting genome-wide expression profiles. Proc. Natl. Acad. Sci. U S A *102*, 15545–15550.

Tarpey, P.S., Behjati, S., Cooke, S.L., Van Loo, P., Wedge, D.C., Pillay, N., Marshall, J., O'Meara, S., Davies, H., Nik-Zainal, S., et al. (2013). Frequent mutation of the major cartilage collagen gene COL2A1 in chondrosarcoma. Nat. Genet. *45*, 923–926.

TCGA. (2017). Comprehensive and integrated genomic characterization of adult soft tissue sarcomas. Cell *171*, 950–965.e28.

Therneau, T.M., and Grambsch, P.M. (2000). Modeling Survival Data: Extending the Cox Model (Springer Science+Business Media, LLC).

Varela, I., Tarpey, P., Raine, K., Huang, D., Ong, C.K., Stephens, P., Davies, H., Jones, D., Lin, M.L., Teague, J., et al. (2011). Exome sequencing identifies frequent mutation of the SWI/SNF complex gene PBRM1 in renal carcinoma. Nature *469*, 539–542.

WHO. (2013). WHO Classification of Tumours of Soft Tissue and Bone, Fourth Edition (WHO).

Zack, T.I., Schumacher, S.E., Carter, S.L., Cherniack, A.D., Saksena, G., Tabak, B., Lawrence, M.S., Zhsng, C.Z., Wala, J., Mermel, C.H., et al. (2013). Pan-cancer patterns of somatic copy number alteration. Nat. Genet. *45*, 1134–1140.

STAR★METHODS

KEY RESOURCES TABLE

REAGENT or RESOURCE	SOURCE	IDENTIFIER
Chemicals, Peptides, and Recombinant Proteins		
MLH1 antibody	Agilent Technologies	cat. NCL-L-MLH1; RRID: AB_10555424
MSH2 antibody	Agilent Technologies	cat. M363901-2; RRID: AB_2631353
MSH6 antibody	Agilent Technologies	cat. M364601-2
PMS2 antibody	BD Biosciences	cat. 556415; RRID: AB_396410
ATRX antibody	Sigma-Aldrich	HPA001906; RRID: AB_1078249
Telomere FISH	Agilent Technologies	K532511-8
Schiff's fuchsin-sulphite reagent	Sigma-Aldrich	S5133
Deposited data		
WGS data	This study	EGA: EGAD00001004162
Methylation array data	This study	ArrayExpress: E-MTAB-6961
RNA seq data	This study	EGA: EGAD00001004439
Software and algorithms		
CaVEMan	(Varela et al., 2011)	https://github.com/cancerit/CaVEMan
cgpPindel	(Raine et al., 2015)	https://github.com/cancerit/cgpPindel
BRASS	(Nik-Zainal et al., 2016)	https://github.com/cancerit/BRASS
ASCAT NGS	(Van Loo et al., 2010)	https://github.com/cancerit/ascatNgs
Battenberg		https://github.com/cancerit/cgpBattenberg
GISTIC 2.0	(Mermel et al., 2011)	http://portals.broadinstitute.org/cgi-bin/cancer/publications/pub_paper.cgi?mode=view&paper_id=216&p=t
Telseq	(Ding et al., 2014)	https://github.com/zd1/telseq
Mutational signatures	(Alexandrov et al., 2018)	https://uk.mathworks.com/matlabcentral/fileexchange/38724-sigprofiler
dndSCV	(Martincorena et al., 2017)	https://github.com/im3sanger/dndscv
CIVIC database	(Griffith et al., 2017)	https://civicdb.org/home
Genie database	(AACR Project GENIE Consortium, 2017)	http://www.aacr.org/Research/Research/Pages/aacr-project-genie.aspx#.WyD4IjMzq34
MSKCC hotspots database	(Chang et al., 2016)	http://cancerhotspots.org/
Encode blacklist	(Encode Project Consortium, 2012)	https://www.encodeproject.org/annotations/ENCSR636HFF/
ExAC database	(Lek et al., 2016)	http://exac.broadinstitute.org/
Variant Effect Predictor	(McLaren et al., 2016)	https://www.ensembl.org/info/docs/tools/vep/index.html
Recurrent rearrangements	This paper	https://github.com/UCL-Research-Department-of-Pathology/RETREAD
Chromothripsis identification	This paper	https://github.com/UCL-Research-Department-of-Pathology/CODER
Copy number signatures	This paper	https://github.com/UCL-Research-Department-of-Pathology/CONUSIG
Mutation triaging	This paper	https://github.com/UCL-Research-Department-of-Pathology/Triagen
Rearrangment signatures	(Nik-Zainal et al., 2016), this paper	https://github.com/UCL-Research-Department-of-Pathology/RESIN
Timing whole genome doubling	(Dentro et al., 2017)	https://github.com/galder-max/USARCtiming
HISAT2	(Kim et al., 2015)	https://github.com/infphilo/hisat2
Stringtie	(Pertea et al., 2015)	https://github.com/gpertea/stringtie
Mutect2	(Van der Auwera et al., 2013)	https://software.broadinstitute.org/gatk/download/auth?package=GATK-archive&version=3.8-1-0-gf15c1c3ef
Lumpy	(Layer et al., 2014)	https://github.com/arq5x/lumpy-sv
ASCAT	(Van Loo et al., 2010)	https://github.com/Crick-CancerGenomics/ascat

CONTACT FOR REAGENT AND RESOURCE SHARING

Further information and requests for resources and reagents should be directed to the Lead Contact, Nischalan Pillay (n.pillay@ucl.ac.uk).

EXPERIMENTAL MODEL AND SUBJECT DETAILS

Patient Samples

Patient tissues and data originated from the Royal National Orthopaedic Hospital biobank, pathology archives and London Sarcoma Service databases. Patient samples were obtained from the Stanmore Musculoskeletal Biobank, a satellite of the UCL/UCLH Biobank (HTA Licence Number 12055), which was approved by the National Research Ethics Committee (reference 15/YH/0311). This specific study was approved by the NHS Health Research Authority (REC reference 16/NW/0769). Informed consent was obtained from all patients.

Case Selection

The pathology archives were searched for sarcomas ICD coded (http://www.who.int/classifications/icd/en/) as undifferentiated sarcoma, pleomorphic sarcoma or spindle cell sarcoma NOS. Only cases where both consent and frozen tissue and matching germline material were available were included. A total of 61 cases were identified where adequate nucleic acid was available. Four cases were excluded by pathology review (R.T and N.P) and immunohistochemistry profiling as they represented other sarcoma types. A further four cases were excluded on analysis of WGS results as they bore molecular hallmarks of other sarcoma types (viz. dedifferentiated liposarcoma, malignant peripheral nerve sheath tumor and malignant solitary fibrous tumor). A total of 53 cases of undifferentiated/unclassified sarcoma were included for further study. Formalin fixed, paraffin embedded tissue blocks of the tumors that were sequenced were used for immunohistochemical and image cytometry analysis.

METHOD DETAILS

Tissue Processing and DNA Extraction

Tumor samples were retrieved from liquid nitrogen stores, embedded in Tissue-Tek OCT and sectioned on a cryostat. For each sample, an initial 5 μm hematoxylin and eosin (H&E) stained section was cut. Microscopic examination of tumor type and tumor cellularity was estimated by a pathologist (N.P). A minimum tumor content of 50% was required for inclusion in the study. Some cases required macrodissection to enrich for tumor content. Twenty-five sections of 20 μm thickness was then collected with a final H&E for confirmation of uniformity of tumor content.

DNA was extracted using an automated magnetic bead extraction and purification system according to the manufactures' protocols (Prepito DNA Tissue10 Kit, Perkin Elmer Ltd, Bucks,UK). DNA from blood was obtained using a column based system (Qiamp DNA Blood Maxi kit, Qiagen,Manchester,UK). DNA concentration and quality were assessed by a fluorometric assay (Picogreen, Thermofisher Scientific,Paisley,UK) and a PCR assay followed by gel electrophoresis. Only DNA that was of suitable concentration (minimum 500 ng total) and was amplifiable were used for whole genome sequencing.

Whole-Genome Sequencing Protocol and Data Processing

Whole genome sequencing was performed on samples on the XTen instrument (Illumina,Chesterford,UK) according to the manufacturers protocol using 150 bp, paired-end libraries with a PCR free workflow. The average coverage of tumors was at least 70X and of normal DNA at least 30X. For classification of genome complexity there was no association between molecular subgroup and tumor purity (Kruskal Wallis test, p=0.13), suggesting that the relatively low number of mutations in the mutLo-rearrLo subgroup is not an artefact of normal tissue contamination of the tumor specimens.

Targeted Sequencing Protocol and Data Processing

Genomic DNA was extracted from formalin fixed paraffin embedded tissues. Samples were chosen based on high tumor content and cellularity.

We designed a DNA target-enrichment design (SureSelect, Agilent Technologies, Santa Clara, CA, USA). We selected 350 genes implicated in cancer and/or sarcoma based on the whole genome sequencing results to serve as a validation assay, identify cancer genes not previously implicated in sarcoma and to assess the frequency of the recurrent mutations.

The bait design also incorporated an Agilent OneSeq 8 Mb copy number backbone evenly spaced across the genome and supplemented by 7000 heterozygous single nucleotide polymorphisms densely tiled across the cancer genes in the assay. DNA and library preparation was carried out as per the manufacturers protocol. Samples were sequenced on a 150 bp paired end high output NextSeq runs. Fastq files were aligned to the human genome reference build GRCh37 using BWA mem.

Hypermutation was defined with a decision boundary of 10 mutations/Mb to account for the large number of subclonal mutations; no rearrangement groups were possible to define for the extension cohort due to the nature of the assay.

RNA Sequencing Protocol and Data Processing

Total RNA was isolated from frozen tissues using the Zymo Direct Zol RNA isolation kit according to manufacturers' recommendations that included the on-column DNase digestion. The quantity and quality of total RNA was assessed by NanoDrop spectrophotometer (Thermo Scientific), Qubit (Thermo Scientific) and Tapestation (Agilent). Only samples with a RIN score >6, high quality spectrophotometer rations and RNA concentration > 250 ng were selected for library preparation.

KAPA Stranded mRNA-Seq kit (Roche- KAPA Biosystems) was used to generate indexed Illumina platform sequencing libraries according to the manufacturer's instructions. Equimolar amounts of libraries were pooled and sequenced on an Illumina HiSeq 2500 instrument using standard protocols for paired end 100 bp sequencing with a desired sequencing depth of ~60 million paired end reads per library.

Fastq files were aligned to the human genome build GrCh37 using HISAT2 (Kim et al., 2015) and gene expression was quantified using stringtie (Pertea et al., 2015).

Methylation Protocol

600 ng of fresh frozen DNA were bisulfite converted using the Zymo EZ DNA methylation Gold kit (Zymo Research Corp.Irvine,-CA,USA) as per manufacturers recommendations. Bisulfite converted samples were processed and hybridized to the Infinium HumanMethylationEPIC beadchip arrays according to the manufacturer's recommendations.

Methylation intensities were normalized with noob background correction and functional normalization using the minfi funnorm function (Aryee et al., 2014) and converted to beta values for downstream analysis. Sex probes, SNP probes and probes with a detection p-value>0.01 in any sample were removed from the dataset.

Immunohistochemistry Protocol

All staining was performed on the Leica Bond III automated immunostaining platform, with peroxidase blocking and detection carried out using the Leica Bond Polymer Refine DAB kit (Leica, DS9800, Leica Microsystems, Milton Keynes, UK) according to manufacturer's instructions. Dewaxing and epitope retrieval were carried out on board using Leica Bond Dewax (Leica, AR9222) and Leica Epitope Retrieval solution 1 or 2 (Leica, AR9961, AR9640). Peroxide block (as per kit) was performed for 5 minutes at ambient temperature prior to primary antibody application. All primary antibodies were diluted in Leica Bond Primary Antibody Diluent (Leica, AR9352) and applied for 30 minutes at ambient temperature. Rabbit-anti-mouse post-primary and anti-rabbit polymer (as per kit) were sequentially applied for 20 minutes each before detection with DAB and counterstaining with hematoxylin.

MLH1

MLH1 (Leica, mouse monoclonal ES05, cat. NCL-L-MLH1): diluted 1/200. Epitope retrieval: ER2 (high pH), 40 minutes, 99°C.

MSH2

MSH2 (Agilent Technologies, mouse monoclonal FE11, cat. M363901-2): diluted 1/50. Epitope retrieval: ER2 (high pH), 20 minutes, 99°C.

MSH6

MSH6 (Agilent Technologies, rabbit monoclonal EP49, cat. M364601-2): diluted 1/50. Epitope retrieval: ER2 (high pH), 30 minutes, 99°C.

PMS2

PMS2 (BD Biosciences, mouse monoclonal A16-4, cat. 556415): diluted 1/300. Epitope retrieval: ER2 (high pH), 40 minutes, 99°C.

ATRX (Sigma-Aldrich, rabbit polyclonal HPA001906): diluted 1/500. Epitope retrieval: ER2 (high pH), 20 minutes,99°C.

Fluorescence *In-Situ* Hybridization Protocol

The alternative lengthening of telomere phenomenon was investigated in the USARC cohort by telomere specific fluorescent in-situ hybridization using previously described methods. In brief, deparaffinized sections were pre-treated by pressure cooking for 5 minutes and subsequently incubated in pepsin solution at 37°C for 50 minutes. Probes (Telomere PNA FISH – FITC; K532511-8; Agilent Technologies LDA UK Limited, Cheshire, UK) were applied to tissue sections and denatured at 72°C, and followed by hybridization overnight at 37°C. After hybridization, the sections were washed and mounted using 4′,6-diamidino-2-phenylindole with coverslips. The telomere phenotype was determined using a published method (Koelsche et al., 2016). This was supplemented by assessing area of fluorescent intensity using the Olympus Cell imaging software in a minimum of 10 tumor cells per case.

Image Cytometry Protocol

Based on a modified protocol of Hedley et al. (1983), 50 μm FFPE sections from USARC samples were deparaffinized and rehydrated. Nuclear suspensions were obtained through cytoplasmic digestion using protease type VIII (Sigma P5380). Samples were filtered, cytospun, and subjected to DNA hydrolysis (5 M HCl) and Feulgen staining (Schiff's fuchsin-sulphite reagent; Sigma S5133). DNA ploidy was measured using the Fairfield ploidy system. A histogram with a DNA index was produced for each sample by calculating the integrated optical density.

QUANTIFICATION AND STATISTICAL ANALYSIS

Somatic Mutation Triaging

Mutations were called using CaVEMan (Varela et al., 2011) and cgpPindel (Raine et al., 2015) for whole-genome sequencing. Only mutations that had median assembly score (ASMD) $\geq$ 140 and median clipped bases (CLPM)=0 were considered reliable mutations. For targeted sequencing, mutations were called using Mutect2 from GATK v3.8 (Van der Auwera et al., 2013). Mutations were prioritized after annotating with VEP (McLaren et al., 2016) as follows:

1 Variants observed in the CIVIC (Griffith et al., 2017), Sanger, Genie (AACR Project GENIE Consortium, 2017) or Memorial Sloane Kettering Cancer Center cancer hotspots database (MSKCC) were categorized as "High confidence".
2 Variants overlapping the Encode Blacklist (Encode Project Consortium, 2012), or that were unidirectional, or were seen in >1.5% of germline reads were categorized as "Unreliable".
3 Silent variants were retained.
4 Variants observed in the ExAC database (Lek et al., 2016) were categorized as "Unreliable".
5 Variants observed in >1 patient wass categorized as "Medium confidence", otherwise "Low confidence".
6 "Medium confidence" variants were re-categorized as "Low confidence" if CADD score <20, IMPACT=LOW or IMPACT=MODIFIER, while "Low confidence" variants were re-categorized as "Medium confidence" if CADD score>20, IMPACT=HIGH or MODERATE or clinsig=pathogenic.

Potential driver mutations were those defined as "High confidence" or "Medium confidence" (see below).

Germline Mutation Triaging

Germline variants were annotated using VEP. Potentially pathogenic variants were restricted to those in coding regions, including splice region variants. Variants were considered pathogenic if their clinical significance was pathogenic, their impact was high or their CADD score was $\geq$30. Additionally, any variant must have passed the ExAC quality filter.

Rearrangement Triaging

Rearrangements were called using BRASS (Nik-Zainal et al., 2016) for whole-genome sequencing, and Lumpy for targeted sequencing. Stringent BRASS calls were defined as those that were able to be locally assembled at base pair resolution and were not observed in the germline.

Rearrangements were then classified as functional (Disruptive or Fusion), or not (Unknown significance or Benign) using BRASS fusion flags as follows:

1 UTR-UTR, single intron or ambiguous flags were classified as "Unknown significance".
2 Fusion flags with different reading frame fusion between an exon and intron, those within the same gene in different regions, those that lead to truncation, those that are a rearrangement between an intron and an intergenic region, and those that are in an opposite orientation between and intron and an exon are classified as disruptive.
3 Those with no predicted fusion, but which have an intronic or exonic breakpoint are classified as disruptive. Otherwise they are classified as benign.
4 In-frame fusions are classified as fusions.
5 Driver disruptions are identified as those that occur in a COSMIC TSG and are not in the UTR.
6 Driver fusions are categorized as those that include a COSMIC fusion gene, and the fusion partner is also included in COSMIC.

Potential driver rearrangements were identified from the functional rearrangements by cross-referencing with the COSMIC database of known cancer driver genes. Out of 452 predicted fusions involving two genes with unambiguous reading frames, 158 predicted in-frame fusions were identified in 47 samples involving 14 cancer genes (16), none of which were recurrent. Of the 14 putative fusions involving known cancer genes, none involved a known fusion partner and no recurrent novel predicted fusion genes were discovered. Moreover, further interrogation of the predicted breakpoint regions revealed that 39% of them directly overlap a known fragile site (Bignell et al., 2010).

Copy-Number Calling

Allele specific copy number and ploidy were called using ASCAT NGS (Van Loo et al., 2010) for whole-genome sequencing, and ASCAT for targeted sequencing. Gene level driver copy number aberrations were identified as such:

Amplification

Minimum total copy number across the gene > $2^{1.3}\times$ ploidy in a known cancer amplified gene (COSMIC).

Homozygous Deletion

Any region within a known tumor suppressor gene (COSMIC) has allele specific copy number = {0,0}.

Loss of Heterozygosity

Any region within a known tumor suppressor gene (COSMIC) has allele specific copy number = {0, $\geq$1} or {$\geq$1,0}.

Recurrent Rearrangements

Genomic regions containing clustered breakpoints in whole-genome sequencing were found by binning the human genome into 1 Mb bins and counting the number of samples with >1 rearrangement breakpoint as identified by stringent BRASS calls (described above) in each (Peifer et al., 2015). To determine a suitable threshold above which to call recurrence significant, 10,000 Monte Carlo simulations of random breakpoint partners were performed as follows:

Let p be the probability of breakpoint partners being on the same chromosome (determined empirically from the dataset). Then:

$$n \sim 1 + Bin(1-p),$$

where n is the number of chromosomes involved in the rearrangement.

Let s_c be the start position of the cth chromosome from set $\boldsymbol{C}$={1,2,…,X,Y}, and e_c be the end position.

Then draw n chromosomes from $\boldsymbol{C}$ with probability. $\{e_1 - s_1, e_2 - s_2, \ldots, e_X - s_X, e_Y - s_Y\} / \sum_{i \in \boldsymbol{C}} e_i - s_i$.

If n=2, then:

$$B_c \sim U(s_c, e_c),$$

where B_c are the breakpoints drawn from the cth chromosomes.

If instead n=1, then:

$$D \sim Exp(1/\mu_c),$$

where D is the distance between the two breakpoints, and μ_c is the mean distance between breakpoints on the cth chromosome (determined empirically from the dataset). Then:

$$B_{1,c} \sim U(s_c, e_c - D),$$

and:

$$B_{2,c} = B_{1,c} + D,$$

where $B_{1,c}$ and $B_{2,c}$ are the 1st and 2nd breakpoint on chromosome c.

If, $\boldsymbol{O}$ is the set of number of rearrangements observed in all samples in the dataset, then rearrangements are simulated N times as:

$$N \sim Gamma(\boldsymbol{k}, \boldsymbol{\theta})).$$

where k and θ are the shape and scale parameters of the gamma distribution, which are estimated from $\boldsymbol{O}$ by maximum likelihood (fitdistr, R MASS package). For each Monte Carlo simulation, x simulated samples are generated, where x matched the number of samples in the dataset.

For a genomic bin with i observed samples with breakpoints, the p-value is then (m+1)/(M+1), where m is the number of simulations with observed samples >i, and M is the number of simulations, here 10,000 . Q-values are then calculated as:

$$q_i = \min_{i \le j \le M} \frac{M \pi_0 P_j}{j}$$

where:

$$\pi_0 = \min\left(1, \frac{2}{M} \sum_{i=1}^{M} P\right)_i,$$

where P are the ordered p-values. Any Q-value <0.2 was considered significant.

Recurrent Copy Number Alterations

Recurrent copy number alterations from whole-genome sequencing at a gene level (-genegistic) were identified using GISTIC 2.0 (Mermel et al., 2011) with a broad analysis (-broad) and arm level peel off (-armpeel), a threshold for deletions and amplifications of 0.25 (-ta,-td), a threshold for broad events of 0.98 (-brlen). The confidence level for calculating driver regions was 0.90 (-conf). Marker-level copy number data was collapsed to gene-level data using the extreme method (-gcm).

Chromothripsis Identification

Regions of chromothripsis were identified using criteria outlined in Korbel and Campbell (2013), namely through clustering of breakpoints, randomness of DNA fragment joins and randomness of DNA fragment order. Chromothripsis was interrogated in sliding windows of 3 Mb, with a spacing of 100 kb. Only chromosomes with >30 breakpoints were considered. A Kolgomorov-Smirnov test was performed on distances between breakpoints of a whole chromosome (not a sliding window) against the exponential distribution with mean equal to the mean breakpoint distance, a p-value threshold <0.05 was used as Korbel and Campbell (2013) state chromothriptic chromosomes have a strong departure from a random distribution. A goodness of fit test was performed on the counts of {HH, HT, TH, TT} joins with a null distribution of {0.25, 0.25, 0.25, 0.25} to test for random DNA joins in sliding windows. A monte carlo simulation of 1000 draws of two breakpoints was performed on sliding windows to test random order, where the

p-value was the proportion of simulations where $|i_{1,s} - i_{2,s}| > |i_{1,d} - i_{2,d}|$ where $i_{1,s}$ is the ordered index of the first breakpoint in the simulated pair of breakpoints, and $i_{1,d}$ is the ordered index of the first breakpoint of a rearrangement pair in the dataset. For random joins and random order a looser threshold of p>0.8 was used, as these tests are aiming to accept the null rather than reject it, and in particular Korbel and Campbell (2013) state that the random order of breakpoints is not entirely random, but more random than a scenario of independent structural rearrangement. If two of the three tests indicated chromothripsis, the window was designated as a potentially chromothriptic event. Note that these tests are able to identify events that do not behave like classical chromothripsis (oscillating between two copy number states, one of which is LOH), but share the hallmarks of chromothripsis. A strict definition of two copy number states was not employed as it precludes copy number alteration preceding or subsequent to chromothripsis, and chromothripsis events have previously been described that involve >>2 copy number states (Behjati et al., 2017; Garsed et al., 2014; Stephens et al., 2011). Likewise, a strict definition requiring oscillating between LOH and non-LOH segments was not incorporated, as it precludes chromothripsis after a WGD event, which is prevalent in our dataset.

Overlapping windows of potentially chromothriptic events were merged. All chromothripsis calls were manually reviewed.

Similar to above, recurrent chromothripsis events were identified by binning the genome into 100 kb bins and counting the number of samples that had a chromothripsis event overlapping each bin.

Telomere Length Estimation

Telomere lengths were estimated from whole genome sequencing reads with a published tool Telseq and using the authors recommended settings (Ding et al., 2014). The weighted average of reads containing at least 7 instances of the telomeric motif TTAGGG in a read group was used to estimate the length.

Mutational Signatures

Mutational signatures were identified using non-negative matrix factorization (NMF) of counts of the triplet context of each mutation in each sample (Alexandrov et al., 2018).

Rearrangement Signatures

Rearrangement signatures were identified using NMF of counts of rearrangements in each sample, classified by type (insertion, deletion, tandem-duplication, translocation), size (1-10 kb, 10 kb-100 kb, 100 kb-1 Mb, 1 Mb-10 Mb, >10 Mb) and whether the rearrangement was clustered or unclustered. The method for determining clustered or unclustered rearrangements was altered from Nik-Zainal et al. (2016); we determine clustered rearrangements as those falling in a piecewise constant fit segment with an average distance between rearrangements less than 0.1x the mean distance between rearrangements across the data set, rather than 0.1x the mean distance between rearrangements in a given sample as is the case in Nik-Zainal et al. (2016). This avoids samples with a majority of rearrangements arising from chromothripsis having none of them called as clustered.

Copy Number Signatures

Copy number signatures were identified from ASCAT allele-specific copy number profiles using NMF. Copy number segments were classified as heterozygous, LOH or homozygous deletions. These were further subclassified by total copy number (0-1=deleted, 2=neutral, 3-4=duplicated, >4=amplified). These were then further subclassified by size of segment (0-0.01 Mb, 0.01-0.1 Mb, 0.1-1 Mb, 1-10 Mb, >10 Mb). This gives a total of 40 mutually exclusive categories a segment can be classified as; 20 LOH categories, 15 heterozygous categories and 5 homozygous deletion categories. NMF was run with ranks 2 through 12 for 1000 runs. The appropriate rank was selected to maximize the consensus silhouette width, the cophenetic distance and the dispersion of clusters. NMF was also run for 1000 runs with ranks 2 through 12 on a randomized version of the data to avoid overfitting.

Following NMF, the deconstructSigs R package (Rosenthal et al., 2016) was used to estimate the exposure of each identified signature in each sample, to reduce overfitting of the exposures to the data.

Validation Cohort

A validation cohort of copy number profiles called by ASCAT were collated from published datasets: 43 chondrosarcoma SNP arrays (Tarpey et al., 2013), 112 osteosarcoma SNP arrays (Behjati et al., 2017) and 203 mixed soft tissue sarcoma SNP arrays (TCGA, 2017). To these we added 15 low grade and assorted sarcoma whole genomes.

Copy number signatures were identified in this cohort in the same manner as for the USARC cohort. Cosine similarities between the USARC signatures and validation signatures were used to determine if signatures were shared across the two cohorts.

Additionally, the validation cohort was scanned for the original USARC copy number signatures using the deconstructSigs R package.

The diversity of copy number signatures identified in each sample for both the validation cohort and the USARC cohort was quantified using Shannon's diversity index as:

$$H = -\sum_{i=1}^{n} p_i \ln p_i,$$

where n is the number of signatures identified in the sample with exposure >0 and p_i is the normalized exposure of the ith signature with exposure >0. Exposures of signatures were normalized to sum to 1.

The probability of the large-scale LOH occurring before WGD is higher than LOH after WGD (P=0.63 and 0.22 respectively, assuming independent chromosome segregation over all possibilities of chromosome segregation events in a model given a diploid or tetraploid cell state).

Survival Analysis

Associations with survival were identified using an accelerated failure time (AFT) model. This model was used because the Cox proportional hazards model's assumption of proportional hazards is violated by several key covariates: resection margins, metastasis status, RB1 mutation status and ATRX mutation status. Patients with only non-primary tumor samples were excluded from analysis. Genetic covariates for a patient with both a primary and metastasis sample were based on the primary sample only. A log-normal AFT model was fit to the log survival times of patients.

An AFT model for overall survival was fit with covariates: size of tumor (mm), resection margins (Complete, Marginal, Incomplete), metastasis status (Metastasis at diagnosis, Metastasis after diagnosis, No metastasis, Unknown) and burden group (mutLo-rearrLo, mutLo-rearrHi, mutHi-rearrLo).

AFT models for metastasis-free survival and progression-free survival were fit with covariates: size of tumor (mm), resection margins (Complete, Marginal, Incomplete) and burden group (mutLo-rearrLo, mutLo-rearrHi, mutHi-rearrLo).

Other models were fit with genetic mutations of *TP53*, *ATRX*, *RB1*, *CDKN2A* and *PTEN* as extra covariates, but none of these were significant. Additional clinical covariates that were previously modelled but found to be non-significant were age at diagnosis, gender, recurrence status. Additional genetic covariates that were previously modelled but found to be non-significant were rearrangement signatures and copy number signatures.

Timing of Whole Genome Duplications

To time whole genome duplications we first inferred the multiplicity of each single nucleotide variant (SNV), i.e. the number of bearing alleles of the mutation (Dentro et al., 2017). Briefly, the most likely multiplicity of an SNV μ_{SNV}, given the purity of the sample ρ, the underlying total tumor copy number $n_{tot,t,SNV}$ and the variant allele fraction of the SNV f_{SNV} is the integer bound by the underlying major allele state of the tumor $n_{major\ ,t,SNV}$, inferred as:

$$\left\langle argmin_{\mu_{SNV}}\left(\left|f_{SNV}\frac{1}{\rho\mu_{SNV}}(\rho n_{tot,t,SNV}+(1-\rho)n_{tot,n,SNV})-1\right|\right)\middle|\mu_{SNV}\in \mathrm{N}_0,\ \mu_{SNV}\le n_{major,t,SNV}\right\rangle$$

where $n_{tot,n,SNV}=2$ is the total copy number of the normal diploid contaminant. As a second step, we separately time whole genome duplications (WGD) in samples having undergone a single WGD (WGDx1) and two WGDs (WGDx2):

1) WGDx1: in regions of the genome represented by 2 copies of the major allele and 0 or 2 copies of the minor allele (annotated 2+0 and 2+2), we count the number of SNVs with a multiplicity of 2 *N2*, i.e. acquired prior to the WGD and the SNVs with a multiplicity of 1 *N1*, i.e. acquired posterior to the WGD. We then time the WGD in relative mutational timing t_{WGD1}:

$$t_{WGD1_{2+0,2+2}}=\frac{N2}{\frac{N1}{2}+N2}$$

2) WGDx2: similarly to WGDx1, in regions of the genome represented by 4 copies of the major allele and 0 or 4 copies of the minor allele (annotated 4+0 and 4+4) we count the number of SNVs with a multiplicity of 4 *N4*, i.e. acquired prior to the first WGD, a multiplicity of 2 *N2*, i.e. acquired prior to the second WGD but posterior to the first, and a multiplicity of 1 *N1*, i.e. late SNVs. We then time the first and second WGDs in relative mutational timing t_{WGD1} and t_{WGD2}, resp.:

$$t_{WGD1_{4+0,4+4}}=\frac{N4}{N4+\frac{N2}{2}+\frac{N1}{4}}$$

$$t_{WGD2_{4+0,4+4}}=\frac{N4+\frac{N2}{2}}{N4+\frac{N2}{2}+\frac{N1}{4}}$$

We compute the 95% confidence intervals from 1,000 bootstrapping of the multiplicities.

Finally, using the same rationale, i.e. early mutations in duplicated regions appear on all duplicated copies, we infer timing of drivers relative to the WGDs from their estimated multiplicities, if they fall in 2+2 or 2+0 regions, and 4+0 or 4+4 regions for WGDx1 and WGDx2, respectively.

To time WGD in TCGA data, we applied the same concepts as outlined, however, we first selected only samples with a major allele $\geq$2 (Figure S7A), i.e. with at least one WGD, and for which our ploidy estimates matched the ploidy estimates in the TCGA publication (TCGA, 2017) (Figure S7B). Timing of WGD events further allowed for testing the possibility of an artefactual origin of the mutLo-rearrLo molecular class. There was no association between either ploidy status or WGD timing with molecular class (Kruskal-Wallis

test, p>0.05). Additionally, there was no significant association between WGD timing, ploidy or normal contamination with rearrangement count in a multivariate regression, as well as no significant interaction between any of the three variables (all p>0.05).

Real-Time Timing

Following the rationale in Gerstung et al. (2017), we scale the relative mutational timing from 0 to the age at diagnosis of the patients. As the acceleration rates are unknown, we did not simulate any acceleration rates. Therefore, our timing estimates might be later than if accounting for acceleration of the clock. So, for a given relative timing of e.g. a whole genome duplication t_{WGD1} in a patient whose age at diagnosis was a, the real-time timing of the WGD rt_{WGD1} becomes

$$rt_{WGD1} = a \times t_{WGD1}$$

and the WGD occurred tbd_{WGD1} years before diagnosis

$$tbd_{WGD1} = a - rt_{WGD1}$$

We verified that the number of spontaneous (C>T)pG were correlated with the age at diagnosis (Figure S7C), as well as strongly correlated with the total number of mutations N_{SNV} (Figure S7D), and N_{SNV} were therefore also correlated with the age of the patients (Figure S7E). Not to lose too many mutations for the real-time estimates of WGD, we used all mutations to derive the relative timing and real-time timing. In our USARC cohort, we verified that the results using only (C>T)pG , where the numbers were sufficient yielded very similar estimates and overall picture (Figure S7F). Using all mutations, we could time WGD in the TCGA exome data, in which the number of mutations is limited and timing using only (C>T)pG becomes inaccurate or even unfeasible.

Gene Set Enrichment Analysis (GSEA)

We performed GSEA using MSigDB collections (Subramanian et al., 2005): c2 KEGG, c2 REACTOME, c5 CC, c5 MF, c6 ALL. To run GSEA, one summary value per gene symbol was used. For each gene, we collapsed the matrix of log2 TPM expression values and the matrix of beta methylation values to the most variable entry. We then selected the 20,000 most variable genes across all samples. We ran two-class GSEA using the signal-to-noise metric with 200 permutations and looked into gene sets with q-value<0.1 (slightly stricter than GSEA recommendation q-value<0.25).

Linear Modelling of Gene Expression

Linear models of gene expression against independent variables were explored for *TP53*, *RB1*, *CDKN2A*, *ATRX*, *PTEN*, *MEN1*, *TERT*, *MSH6*, *MSH2* and *MBD4*. Initial models were parameterized as:

$$\gamma = \beta_0 + \beta_1\pi + \beta_2\chi,$$

where γ is gene expression, π is tumor purity and χ is minimum gene copy number. These models were further expanded to include methylation probe beta values, μ, as dependent variables:

$$\gamma = \beta_0 + \beta_1\pi + \beta_2\chi + \beta_3\mu.$$

The p-values for copy number from the initial models were corrected for multiple testing over 10 genes, while the p-values for methylation probe beta values were corrected for multiple testing over 402 probes.

Statistical Analysis

All statistical analysis was performed using R (R Core Team, 2017). Survival analysis was performed using the survival (Therneau and Grambsch, 2000) and flexsurv (Jackson, 2016) packages. NMF was performed using the NMF (Gaujoux and Seoighe, 2010) package.

DATA AND SOFTWARE AVAILABILITY

Software Availability

Our implementations of methods are available at:

https://github.com/UCL-Research-Department-of-Pathology.
https://uk.mathworks.com/matlabcentral/fileexchange/38724-sigprofiler.
https://github.com/galder-max/USARCtiming.

Data Availability

The accession number for the whole genome sequencing reported in this paper is deposited in EGA database at EMBL-EBI (https://www.ebi.ac.uk/ega/about/access) under accession number EGAD00001004162.

The accession number for the methylation array data reported in this paper is deposited in the ArrayExpress database at EMBL-EBI (www.ebi.ac.uk/arrayexpress) under accession number E-MTAB-6961.

The accession number for the RNA sequencing data reported in this paper is deposited in EGA database under accession number EGAD00001004439.

Cancer Cell, Volume *35*

Supplemental Information

Undifferentiated Sarcomas Develop through Distinct Evolutionary Pathways

Christopher D. Steele, Maxime Tarabichi, Dahmane Oukrif, Amy P. Webster, Hongtao Ye, Matthew Fittall, Patrick Lombard, Iñigo Martincorena, Patrick S. Tarpey, Grace Collord, Kerstin Haase, Sandra J. Strauss, Fitim Berisha, Heli Vaikkinen, Pawan Dhami, Marnix Jansen, Sam Behjati, M. Fernanda Amary, Roberto Tirabosco, Andrew Feber, Peter J. Campbell, Ludmil B. Alexandrov, Peter Van Loo, Adrienne M. Flanagan, and Nischalan Pillay

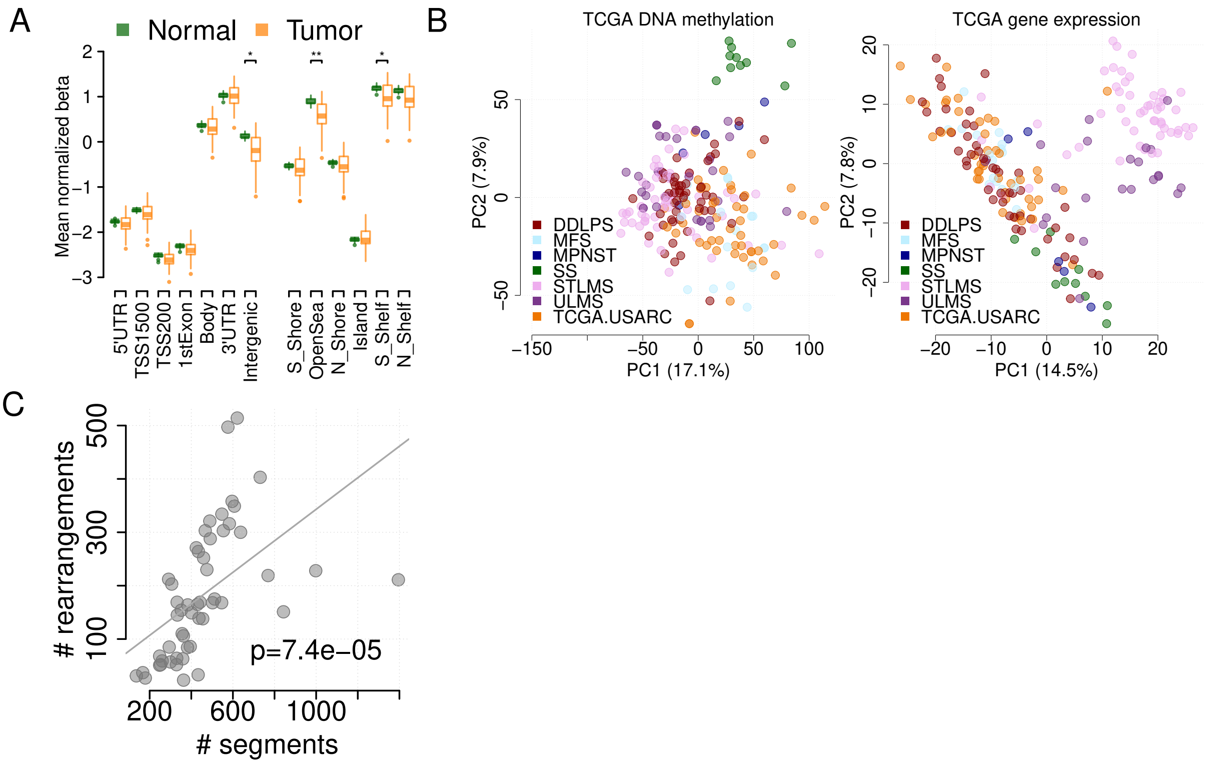

Figure S1. Methylation and RNA patterns in TCGA sarcoma cohort. Related to Figure 1.

(A) Methylation profiling demonstrated large differences between TCGA USARC (orange) and our cohort adjacent normal tissue (green). The pattern of predominant hypomethylation in intergenic and open sea regions observed in USARC was recapitulated here. Boxes show lower quartile, median and upper quartile; lines denote furthest point within 1.5x the interquartile range away from the box; points denote data further than 1.5x the interquartile range away from the box.

(B) Principal components analysis of DNA methylation data (left) or gene expression data (log_{10}(FPKM+1), right) from all sarcoma subtypes in TCGA. Methylation PCs distinguish synovial sarcoma from all other subtypes, and fails to identify distinct USARC subgroups, while RNA PCs distinguish uterine and soft-tissue leiomyosarcoma from all other subtypes, and fail to identify distinct USARC subgroups.

(C) The number of copy number alterations (number of segments) and number of rearrangement breakpoints are strongly correlated (linear regression, β_1=0.3, $p<0.001$).

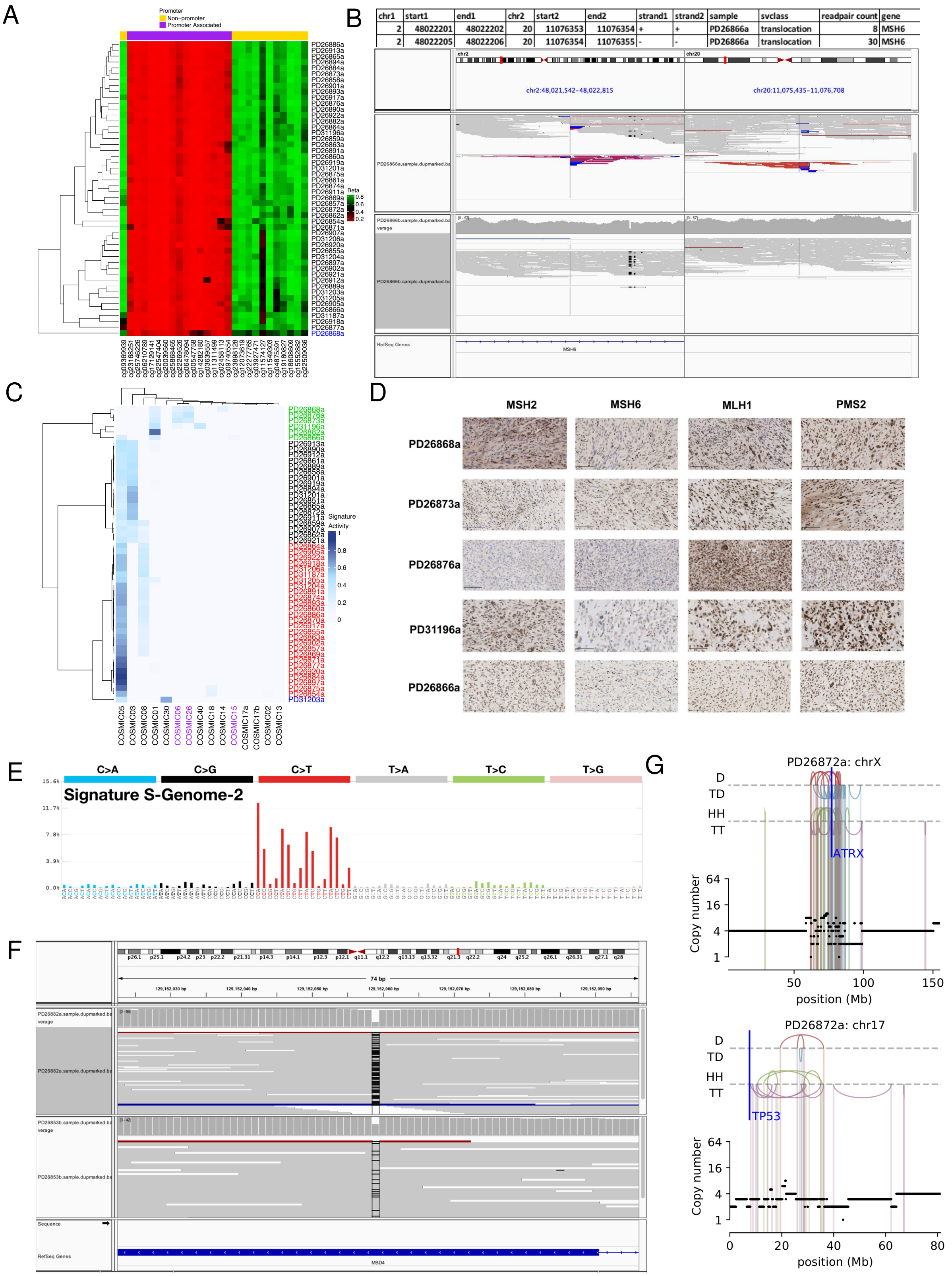

Figure S2. Mismatch repair deficiency and hypermutation in USARC. Related to Figure 1.

(A) Heatmap of beta values of EPIC methylation probes overlapping *MSH2*. PD26868a (blue text) shows relative promoter hypermethylation and gene body hypomethylation compared to all other samples, suggesting *MSH2* silencing through epigenetic regulation.

(B) IGV plot of *MSH6* translocation t(2;20) breakpoints that are predicted to be disruptive.
(C) Mutational signature heatmap. Cosmic mismatch repair deficiency signatures are shown in purple text. Samples are clustered into four groups; hypermutators (green text), homologous recombination deficient (black text), unknown aetiology (red text) and *NTHL1* deficient (blue text).
(D) Mismatch repair protein immunohistochemistry. *MSH2*, *MSH6*, *MLH1* and *PMS2* immunohistochemistry corroborate gene sequencing, mutational signature and methylation findings. Concordant loss of protein expression of *MSH2* and *MSH6* in tumor cells is seen in PD26868a, PD26873a, PD26876a and PD31196a. The *NTHL1* deficient tumor (PD31203a) shows retention of all four mismatch repair proteins. Normal inflammatory cells serve as internal control. Scalebar:100μm.
(E) Sequence context of spectra of mutations (predominantly C>T transitions) indicative of COSMIC signature 30 in sample PD31203a.
(F) PD26882a: IGV browser plot of *MBD4* showing a heterozygous DNA glycosylase domain frameshift mutation (p.L482Wfs*9) in the germline (lower) with loss of heterozygosity in the tumor sample (upper).
(G) Chromothriptic events are observed in regions with key driver genes. Individual rearrangements are shown in top panel (D=deletion, TD=tandem duplication, HH and TT=head-head and tail-tail inversion respectively), total copy number is shown in bottom panel. Genes of interest are highlighted in blue. Translocations are not displayed.

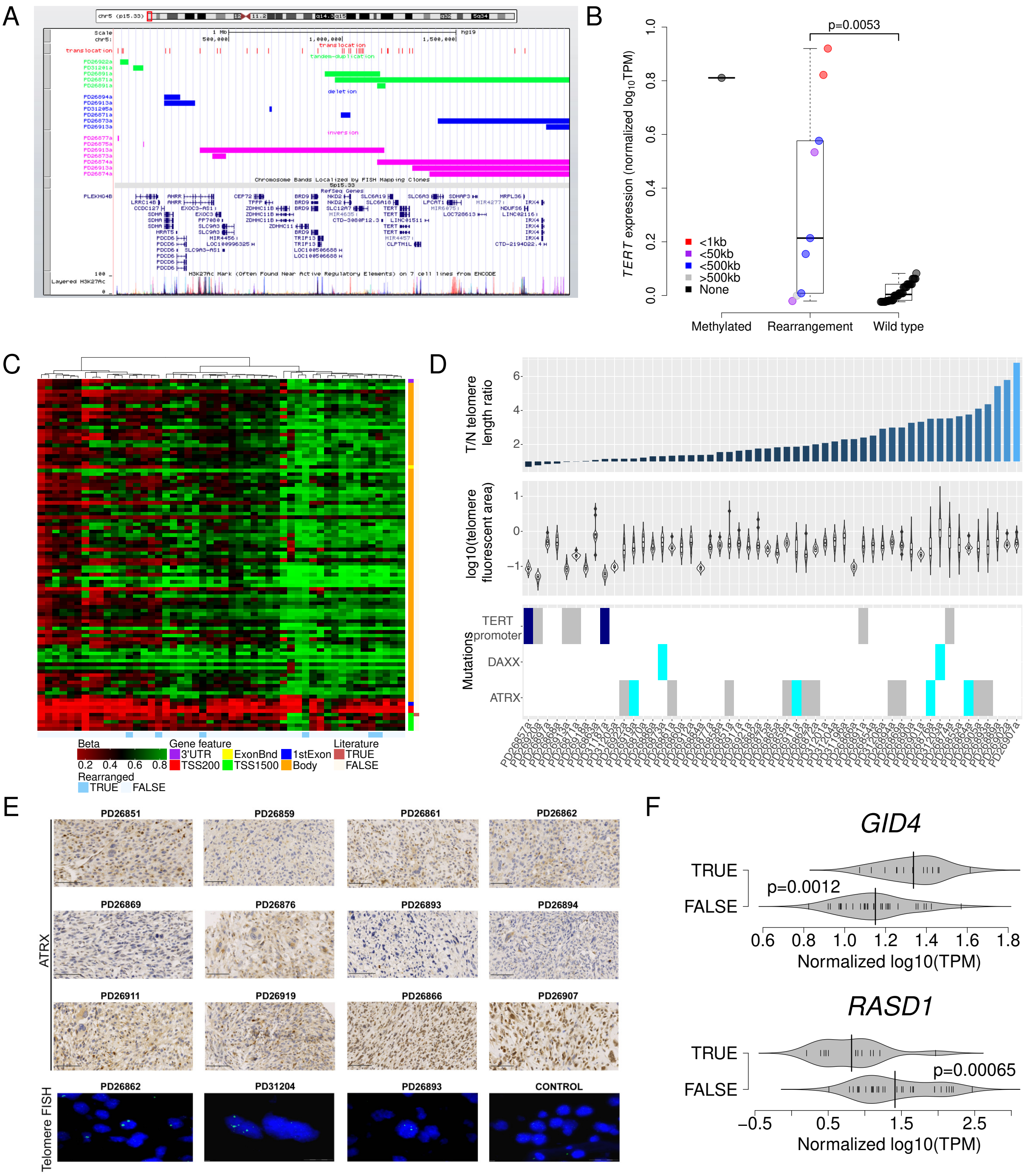

Figure S3. Telomere maintenance pathway. Related to Figure 1.

(A) Browser plot demonstrating the number and spectrum of rearrangements in proximity to *TERT*.

(B) Boxplot of *TERT* expression in samples that have promoter hypermethylation of cg11625005 (Methylated), a rearrangement within 100kb of *TERT* (Rearrangement) or no alterations in *TERT* (Wild Type). Color indicates the distance between the non-TERT rearrangement partner and its closest muscle-specific enhancer. Samples with a rearrangement within 100kb of *TERT* have increased expression of *TERT*, with those that colocalise *TERT* closest to a muscle-specific enhancer (red) having the strongest expression of *TERT*, suggesting activation of *TERT* through enhancer

hijacking. Boxes show lower quartile, median and upper quartile; lines denote furthest point within 1.5x the interquartile range away from the box.

(C) Methylation of probes surrounding *TERT*. X-axis=samples, y-axis=probes. Probe cg11625005 (brown annotation) in PD26857a and PD31187a show relative hypermethylation of the commonly epi-mutated probe cg11625005 in the *TERT* promoter region. Samples with a blue annotation harbor a rearrangement within 100kb of *TERT*.

(D) Tumor:normal telomere ratio estimated from whole genome sequencing. 88.4% of tumor samples show relative telomere lengthening compared to matched normal tissue (upper panel). Violin plots with integrated boxplots indicate telomere FISH analysis data where fluorescent signal area was measured in a minimum of 10 cells (middle panel). *ATRX* and *DAXX* mutations are associated with telomere lengthening (bottom panel). Blue box: promoter epimutation. Grey box: rearrangement. Aqua box: single nucleotide variant or indel.

(E) *ATRX* immunohistochemistry results confirm that *ATRX* mutant samples show loss of protein expression in tumor cells. Representative *ATRX* wildtype samples demonstrating retained nuclear *ATRX* expression (PD26866a, PD26907a). Scalebar 100μm. Representative FISH images of telomere fluorescence demonstrating large, bright signals in tumor cells. Testicular tissue used as a control demonstrates the difference in telomere signal size and intensity.

(F) Genes in the 17p11.2 region that have significantly correlated gene expression and rearrangement status.

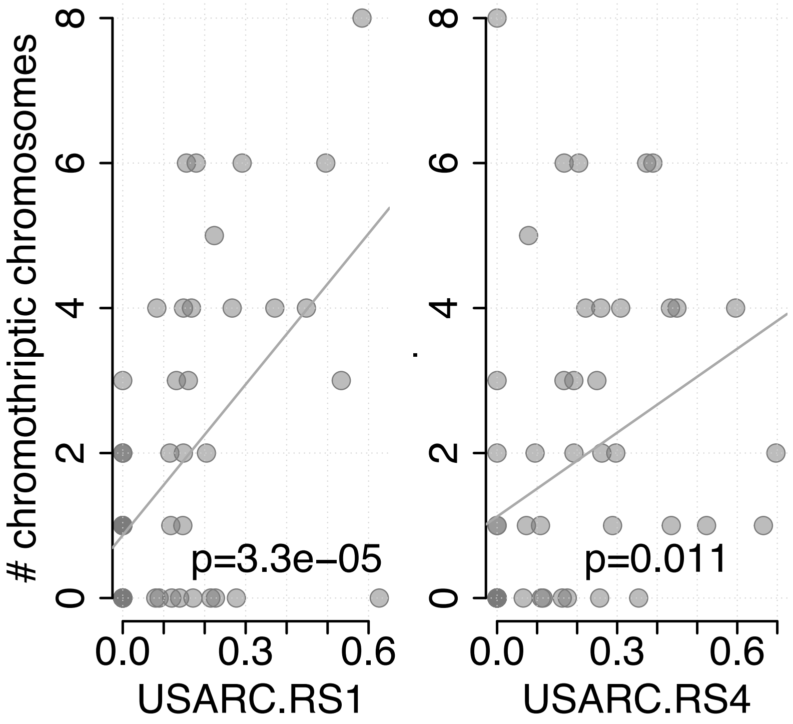

Figure S4. USARC genomic instability correlates. Related to Figure 4.
The number of chromothriptic chromosomes is positively correlated with both the exposure to USARC.RS1, and CNS5.

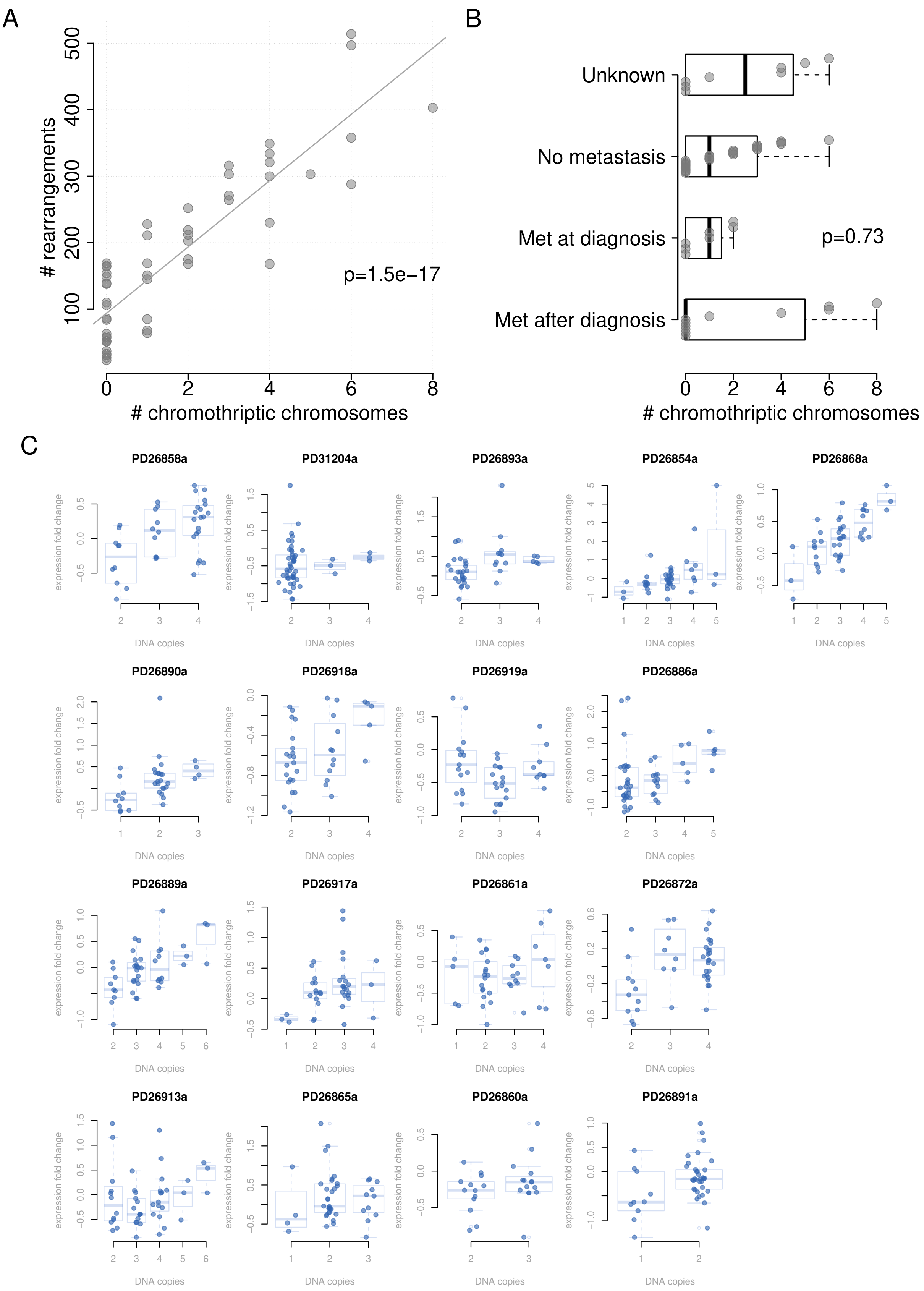

Figure S5. Copy number has a direct effect on gene expression. Related to Figure 5.

(A) The number of chromothriptic chromosomes is correlated with the number of rearrangements and is enriched in the rearrHi group (samples with > 100 rearrangements).

(B) There is no effect of chromothriptic chromosomes on metastasis status (ANOVA, p=0.4). Boxes show lower quartile, median and upper quartile; lines denote furthest point within 1.5x the interquartile range away from the box.

(C) Gene expression fold change compared to all other samples (y-axis) dependent on copy number (x-axis) for multiple samples (panels) displays a trend of increasing gene expression with increasing copy number.

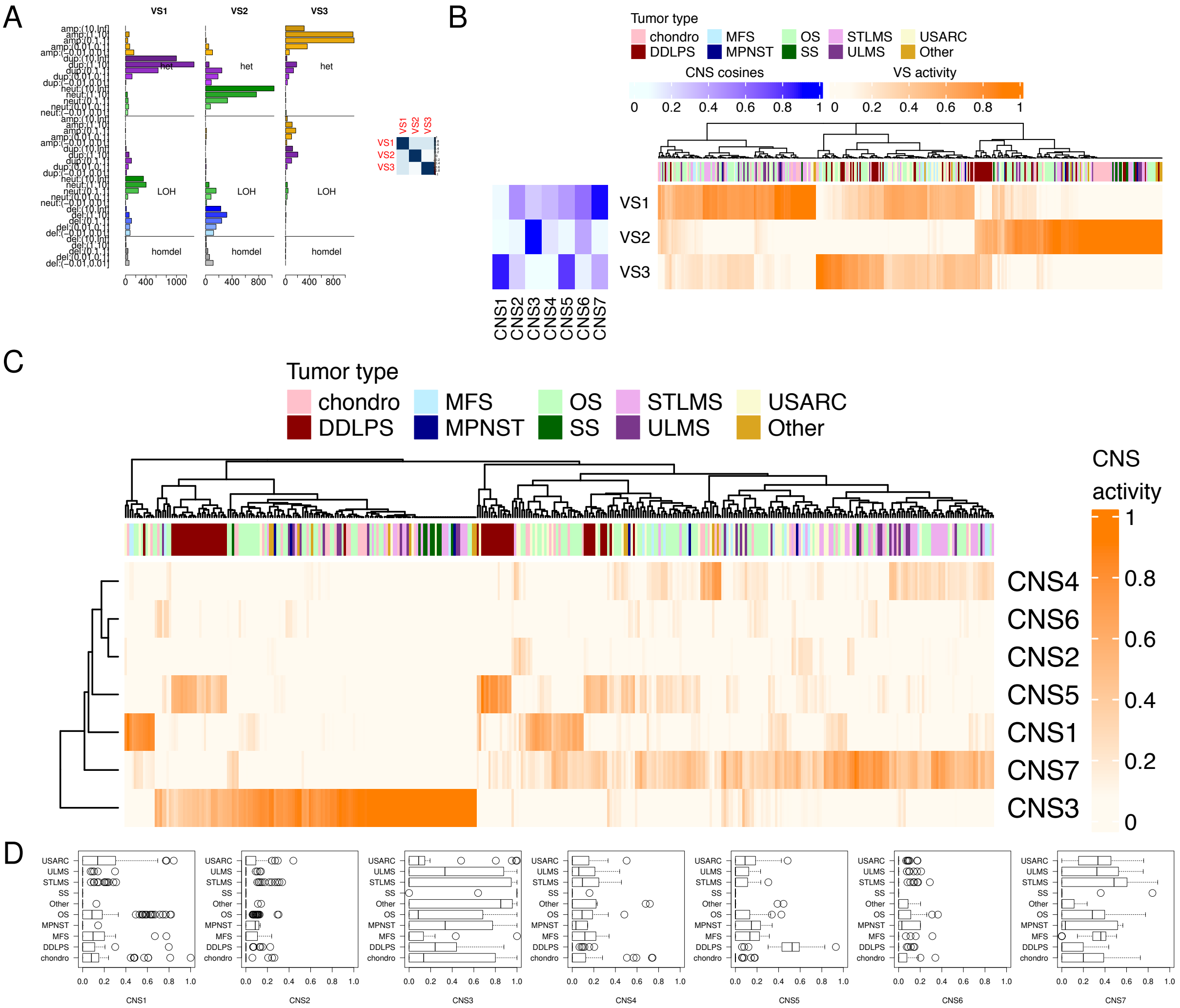

Figure S6. Copy number signature validation. Related to Figure 5.

(A) Three copy number signatures identified in 320 sarcomas of multiple subtypes; 43 chondrosarcoma (chondro), 51 dedifferentiated liposarcoma (DDPLS), 17 myxofibrosarcomas (MFS), 6 malignant peripheral nerve sheath tumor (MPNST), 112 osteosarcoma (OS), 10 synovial sarcoma (SS), 52 soft tissue leiomyosarcoma (SFLMS), 27 uterine leiomyosarcoma (ULMS), 43 undifferentiated pleomorphic sarcoma (UPS), and 12 assorted sarcomas (Other).

(B) Activities of each validation signature in the validation cohort per sample. Cosine similarities between USARC signatures (CNS1-7) and validation signatures (blue heatmap). VS1-3 have all been identified in the USARC cohort.

(C) Predicted activities of CNS1-7 in the validation cohort (TCGA samples and other non-USARC sarcomas) using deconstructSigs.

(D) Boxplot of predicted activities of CNS1-7 in each tumor type. UPS – undifferentiated pleomorphic sarcoma, ULMS – uterine leiomyosarcoma, STLMS – soft tissue leiomyosarcoma, SS – synovial sarcoma, OS – osteosarcoma, MPNST – malignant peripheral nerve sheath tumor, MFS – myxofibrosarcoma, DDLPS – dedifferentiated liposarcoma, chondro – chondrosarcoma, Other – variety of low grade spindle cell sarcomas. Boxes show lower quartile, median and upper quartile; lines denote furthest point within 1.5x the interquartile range away from the box; points denote data further than 1.5x the interquartile range away from the box.

Table S6 – Samples amenable to mutational and genome-duplication timing in the USARC and TCGA datasets. Related to Figure 7.

	USARC	TCGA
Timed	47 (10 CNsig1; 1 CNsig2; 0 CNsig3; 7 CNsig4; 7 CNsig5; 4 CNsig6; 18 CNsig7)	84 (6 CNsig1; 1 CNsig2; 1 CNsig3; 5 CNsig4; 19 CNsig5; 52 CNsig7)
Not timeable	6 (6 CNsig 3)	8 (8 CNsig 5)
Ploidy disagreement	-	47
Signatures not available	-	10

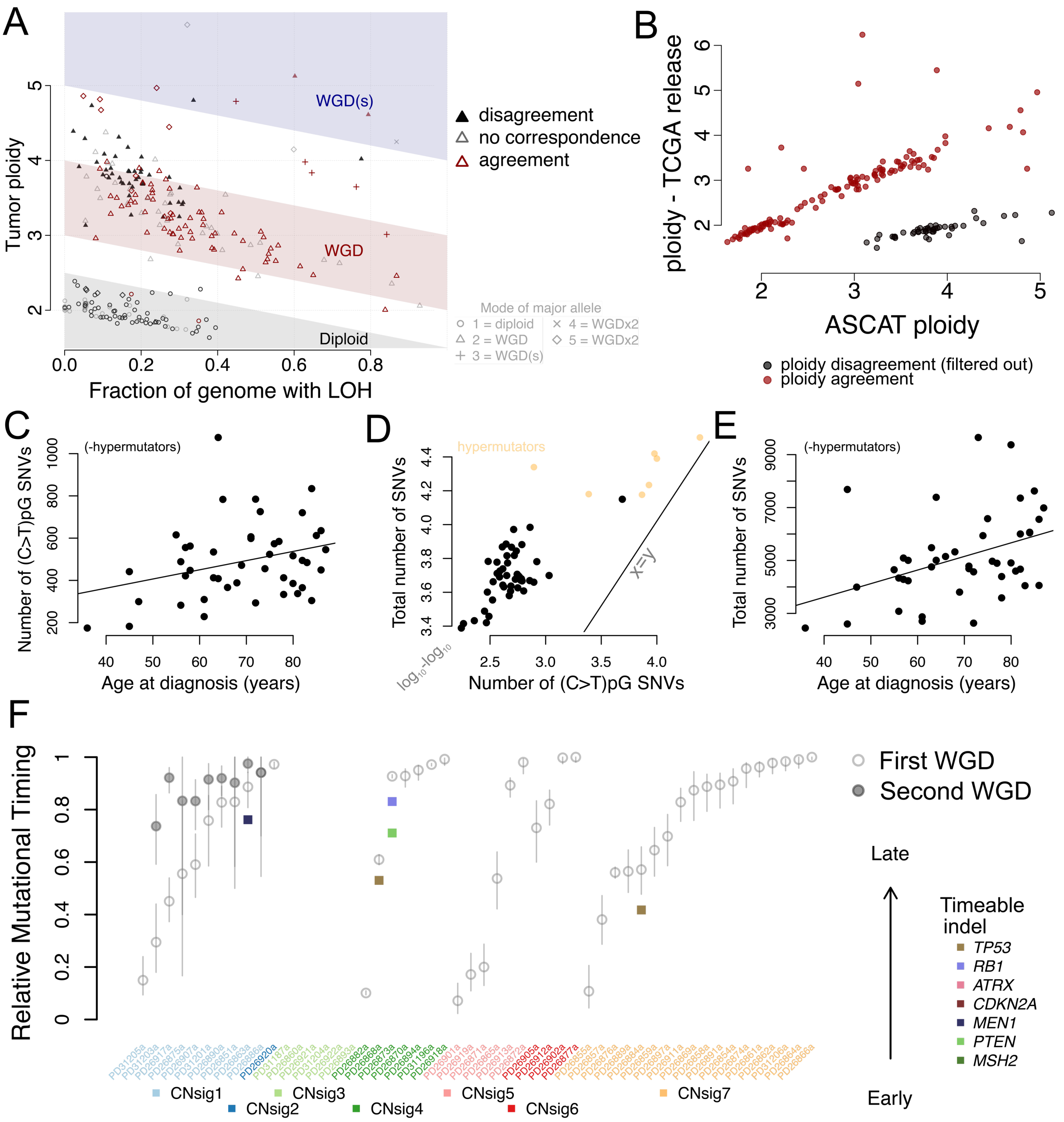

Figure S7. Timing of mutations and genome doubling events. Related to Figure 7.

(A) Ploidy/LOH space for TCGA cohort. Points are colored by whether the ASCAT estimate of the ploidy agrees with that from ABSOLUTE published by TCGA, whereas the shape of the data points relates to their ploidy and represents the mode of the major allele.

(B) TCGA ABSOLUTE ploidy against ASCAT ploidy. Only those samples in agreement (red) were taken further for timing analysis.

(C) Linear relationship between number of (C>T)pG SNVs and age at diagnosis.

(D) Relationship between total number of SNVs and number of (C>T)pG SNVs in samples. Equality line is shown. The gradient of a linear fit is similar to the equality gradient, indicating that timing will be similar using either total SNVs or only (C>T)pG SNVs.

(E) Linear relationship between total number of SNVs and age at diagnosis.

(F) Timing of genome doubling events and driver mutations in the USARC cohort using only (C>T)pG SNVs. Confidence intervals are larger than when using all SNVs (see Figure 5), but the broad picture remains similar. MRCA – most common recent ancestor. Vertical bars: 95% confidence intervals.
